# Supplementary figures and images for: Development of circulating isolates of Plasmodium falciparum is accelerated in Anopheles vectors with reduced reproductive output
Source: PLoS Negl Trop Dis. 2024 Jan 11;18(1):e0011890. doi: 10.1371/journal.pntd.0011890 (PMC10807765; doi:10.1371/journal.pntd.0011890)

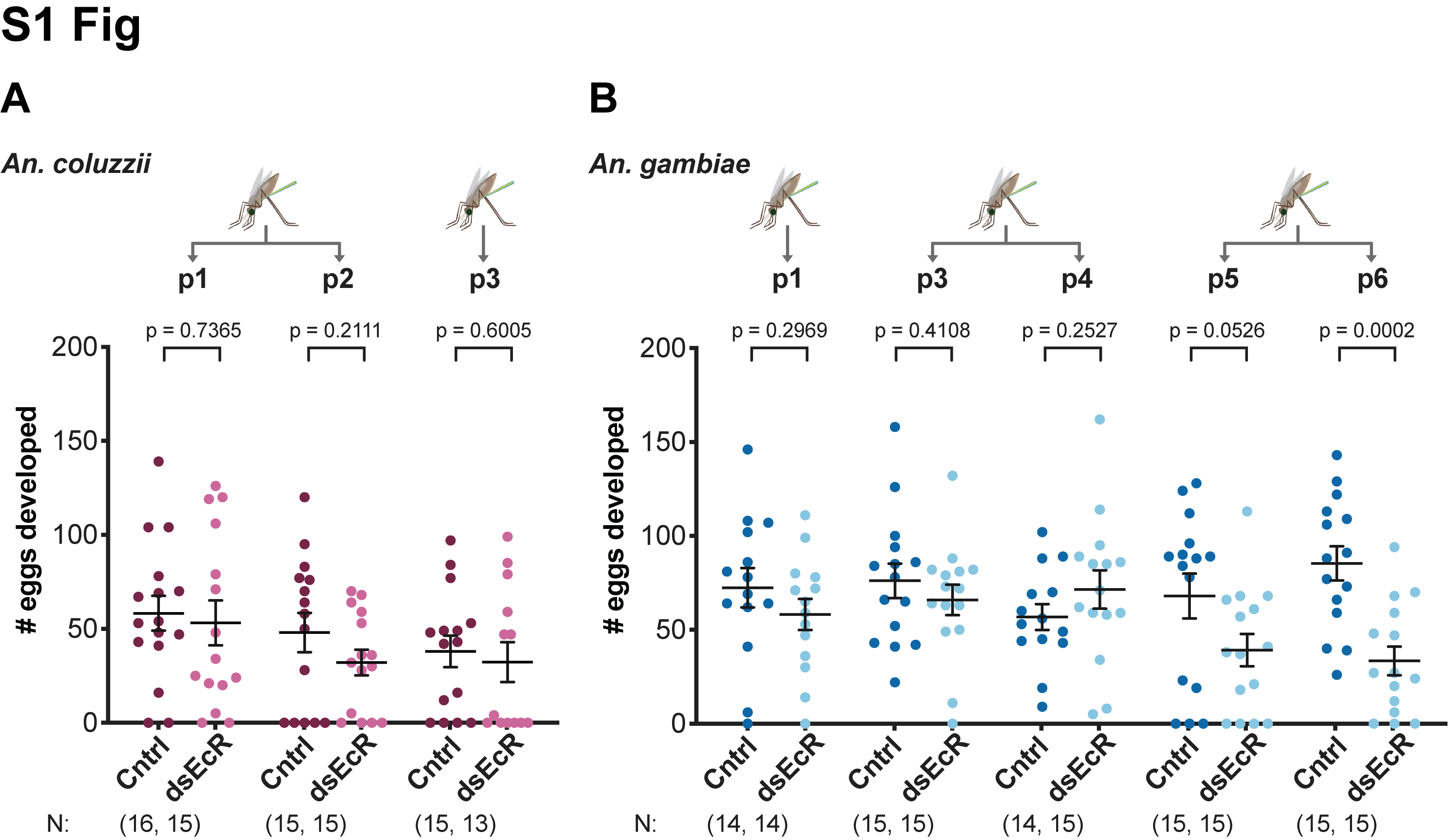

Supplement: S1 Fig — (A-B) The effects of EcR-silencing on egg development across individual infections for (A) An. coluzzii (unpaired t-test and Mann-Whitney) and (B) An. gambiae (unpaired t-test and Mann-Whitney) compared to controls (Cntrl). N = sample size. p# = parasite isolate. (TIF) [file pntd.0011890.s001.tif]

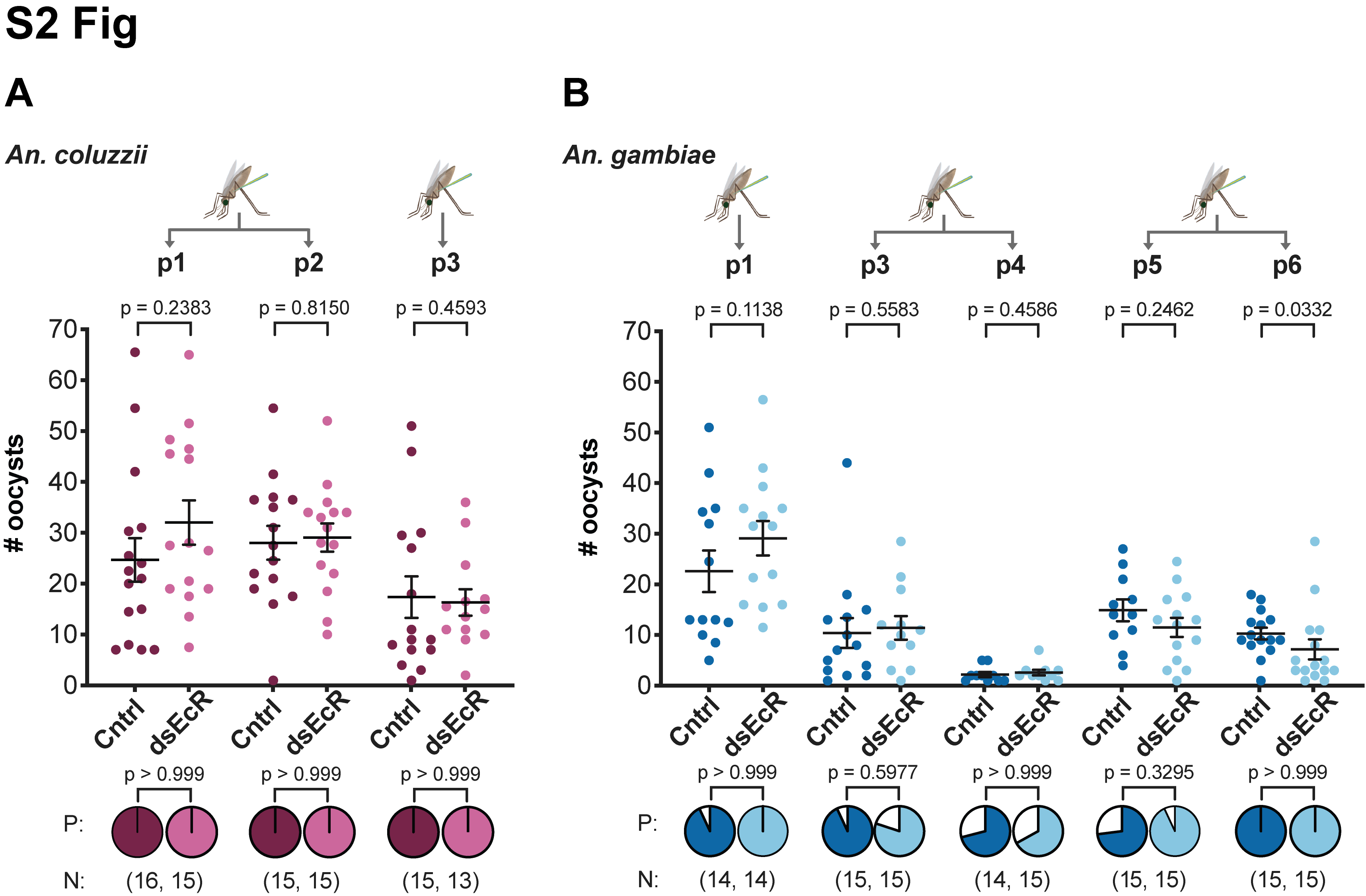

Supplement: S2 Fig — (A-B) The effects of EcR-silencing on the prevalence (Fisher’s Exact) and intensity (unpaired t-test and Mann-Whitney) of oocysts across individual infections for (A) An. coluzzii and (B) An. gambiae, compared to dsGFP-injected (Cntrl) females. P = oocyst prevalence. N = sample size. p# = parasite isolate. (TIF) [file pntd.0011890.s002.tif]

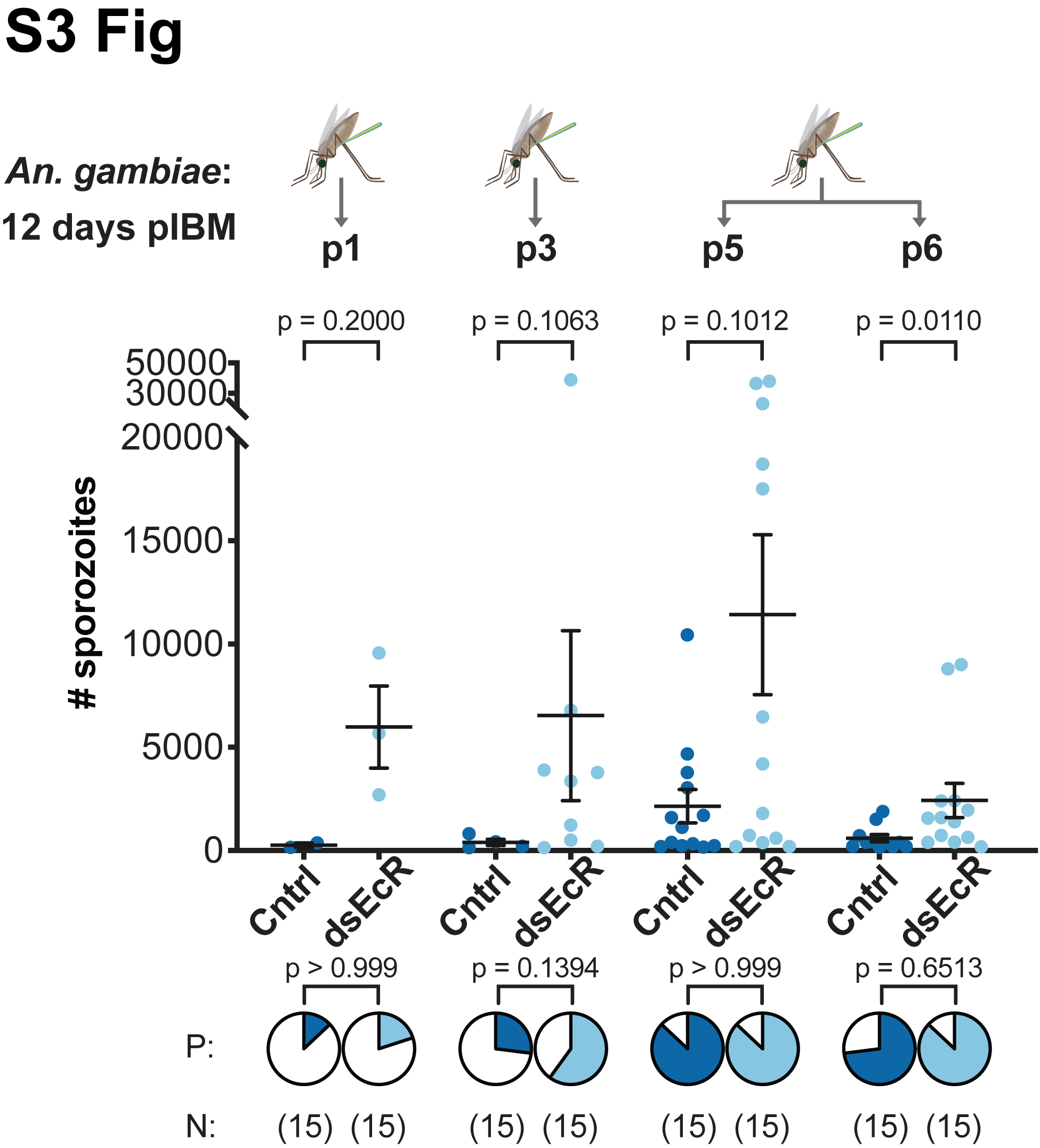

Supplement: S3 Fig — (A) The salivary glands of dsEcR and dsGFP (Cntrl) An. gambiae females were assessed at 12 days post-infected blood meal from infections with parasite isolates p1, p3, p5, and p6. The sporozoite prevalence (Fisher’s Exact) and intensity (Mann-Whitney) for each infection are shown. P = sporozoite prevalence. N = sample size. p# = parasite isolate. (TIF) [file pntd.0011890.s003.tif]

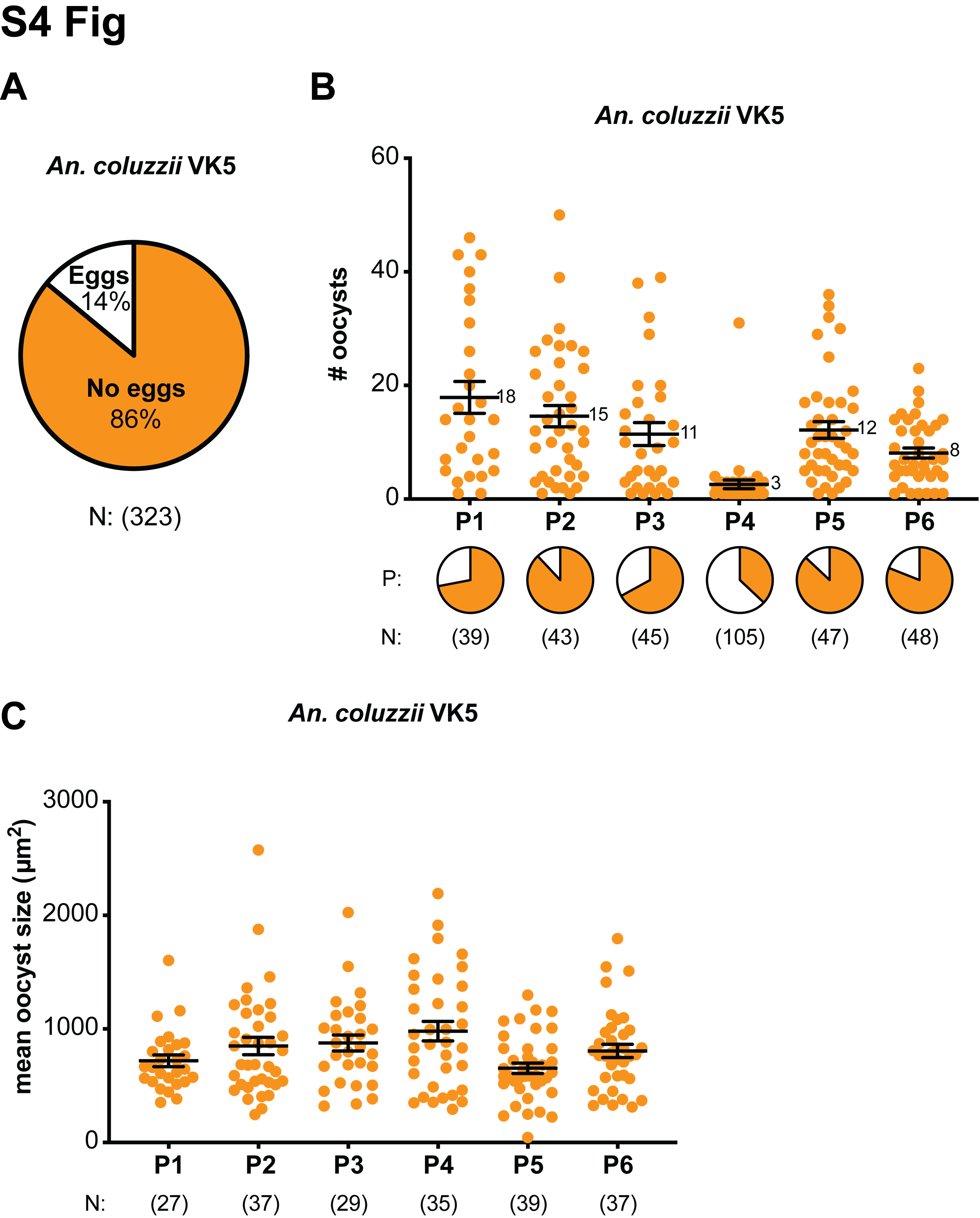

Supplement: S4 Fig — (A) The majority of VK5-An. coluzzii females that were provided a parasite-infected (p1-p6) blood meal, failed to develop any eggs. (B) Oocyst prevalence (P) and intensity for individual infections with VK5-An. coluzzii. (C) Mean oocyst size per VK5-An. coluzzii female for each infection with a different parasite isolate (P#). Mean oocyst sizes are shown for simplicity, but all analyses were done with all individual oocyst measurements nested by mosquito. N = sample size. P# = parasite isolate. (TIF) [file pntd.0011890.s004.tif]
