# Supplementary material for: Development of circulating isolates of Plasmodium falciparum is accelerated in Anopheles vectors with reduced reproductive output
Source: PLoS Negl Trop Dis. 2024 Jan 11;18(1):e0011890. doi: 10.1371/journal.pntd.0011890 (PMC10807765; doi:10.1371/journal.pntd.0011890)
Supplement: Attachment — Submitted filename: PNTD-D-23-01047_REV.pdf [file pntd.0011890.s009.pdf]

PLOS Neglected Tropical Diseases

Impairing reproductive processes in <i>Anopheles</i> vectors accelerates development of circulating isolates of <i>Plasmodium falciparum</i> malaria

--Manuscript Draft--

**Manuscript Number:**	PNTD-D-23-01047
**Full Title:**	Impairing reproductive processes in <i>Anopheles</i> vectors accelerates development of circulating isolates of <i>Plasmodium falciparum</i> malaria
**Short Title:**	<i>Plasmodium</i> growth is linked to mosquito oogenesis in field settings
**Article Type:**	Research Article
**Keywords:**	Anopheles; Plasmodium; malaria; parasite transmission; oocyst growth; oogenesis; vector-parasite interactions; malaria-endemic regions; parasite genetic diversity
**Abstract:**	Anopheles gambiae and its sibling species Anopheles coluzzii are the most efficient vectors of the malaria parasite Plasmodium falciparum. When females of these species feed on an infected human host, oogenesis and parasite development proceed concurrently, but interactions between these processes are not fully understood. Using multiple natural P. falciparum isolates from Burkina Faso, we show that in both vectors, impairing steroid hormone signaling to disrupt oogenesis leads to accelerated oocyst growth and in a manner that appears to depend on both parasite and mosquito genotype. Consistently, we find that egg numbers are negatively linked to oocyst size, a metric for the rate of oocyst development. Oocyst growth rates are also strongly accelerated in females that are in a pre-gravid state, i.e. that fail to develop eggs after an initial blood meal. Overall, these findings advance our understanding of mosquito-parasite interactions that influence P. falciparum development in malaria-endemic regions.
**Additional Information:**	
**Question**	**Response**
**Government Employee**<br><br>Are you or any of the contributing authors an employee of the United States government?<br><br>Manuscripts authored by one or more US Government employees are not copyrighted, but are licensed under a CC0 Public Domain Dedication, which allows unlimited distribution and reuse of the article for any lawful purpose. This is a legal requirement for US Government employees.<br><br>This will be typeset if the manuscript is accepted for publication.	No - No authors are employees of the U.S. government.
**Financial Disclosure**<br><br>Enter a financial disclosure statement that describes the sources of funding for the	Yes

Powered by Editorial Manager® and ProduXion Manager® from Aries Systems Corporation

work included in this submission. Review the submission guidelines for detailed requirements. View published research articles from *PLOS Neglected Tropical Diseases* for specific examples.<br><br>This statement is required for submission and **will appear in the published article** if the submission is accepted. Please make sure it is accurate.<br><br>**Funded studies**<br>Enter a statement with the following details:<br>• Initials of the authors who received each award<br>• Grant numbers awarded to each author<br>• The full name of each funder<br>• URL of each funder website<br>• Did the sponsors or funders play any role in the study design, data collection and analysis, decision to publish, or preparation of the manuscript?<br><br>Did you receive funding for this work?	
Please add funding details.<br>as follow-up to "**Financial Disclosure**<br><br>Enter a financial disclosure statement that describes the sources of funding for the work included in this submission. Review the submission guidelines for detailed requirements. View published research articles from *PLOS Neglected Tropical Diseases* for specific examples.<br><br>This statement is required for submission and **will appear in the published article** if the submission is accepted. Please make sure it is accurate.	This work was funded by the National Institutes of Health (NIH) (R01 AI124165, R01 AI104956) and by a Faculty Research Scholar Award by the Howard Hughes Medical Institute (HHMI) and the Bill & Melinda Gates Foundation (BMGF) (Grant ID: OPP1158190) to FC. FC is a HHMI Investigator. The funders had no role in the study design, data collection, data analysis, data interpretation, decision to publish or preparation of the manuscript. The findings and conclusions herein do not necessarily reflect positions or policies of the NIH, HHMI or BMGF.

**Funded studies**<br>Enter a statement with the following details:<br>• Initials of the authors who received each award<br>• Grant numbers awarded to each author<br>• The full name of each funder<br>• URL of each funder website<br>• Did the sponsors or funders play any role in the study design, data collection and analysis, decision to publish, or preparation of the manuscript?<br><br>Did you receive funding for this work?"	
Please select the country of your main research funder (please select carefully as in some cases this is used in fee calculation).<br>as follow-up to "**Financial Disclosure**<br><br>Enter a financial disclosure statement that describes the sources of funding for the work included in this submission. Review the submission guidelines for detailed requirements. View published research articles from *PLOS Neglected Tropical Diseases* for specific examples.<br><br>This statement is required for submission and **will appear in the published article** if the submission is accepted. Please make sure it is accurate.<br><br>**Funded studies**<br>Enter a statement with the following details:<br>• Initials of the authors who received each award<br>• Grant numbers awarded to each author<br>• The full name of each funder<br>• URL of each funder website<br>• Did the sponsors or funders play any role in the study design, data collection and analysis, decision to publish, or preparation of the manuscript?<br><br>Did you receive funding for this work?"	UNITED STATES - US
**Competing Interests**	The authors declare no competing interests.

Powered by Editorial Manager® and ProduXion Manager® from Aries Systems Corporation

On behalf of all authors, disclose any competing interests that could be perceived to bias this work.<br><br>This statement will be typeset if the manuscript is accepted for publication.<br><br>Review the instructions link below and PLOS NTDs' competing interests policy to determine what information must be disclosed at submission.	
**Data Availability**<br><br>Provide a **Data Availability Statement** in the box below. This statement should detail where the data used in this submission can be accessed. This statement will be typeset if the manuscript is accepted for publication.<br><br>Before publication, authors are required to make all data underlying their findings fully available, without restriction. Review our PLOS Data Policy page for detailed information on this policy. Instructions for writing your Data Availability statement can be accessed via the Instructions link below.	The datasets generated and analyzed in the current study will be available in the Harvard Dataverse repository under the identifier https://doi:10.7910/DVN/AVVLVI

Powered by Editorial Manager® and ProduXion Manager® from Aries Systems Corporation

Click here to access/download;Manuscript;Manuscript.pdf

Title: Impairing reproductive processes in *Anopheles* vectors accelerates development of circulating isolates of *Plasmodium falciparum* malaria

Short title: *Plasmodium* growth is linked to mosquito oogenesis in field settings

Kristine Werling[1], Maurice A. Itoe[1], W. Robert Shaw[1,4], Raymond Dombagniro Hien[2], Bali Jean Bazié[2], Fofana Aminata[2], Kelsey Adams[1], Bienvenu Seydou Ouattara[2], Mathias Sanou[2], Duo Peng[1], Roch K. Dabiré[2], Dari F Da[2], Rakiswendé Serge Yerbanga[2], Abdoulaye Diabaté[2], Thierry Lefèvre[2,3,*], Flaminia Catteruccia[1,4,*]

[1]Department of Immunology and Infectious Diseases, Harvard T.H. Chan School of Public Health, Boston, MA, USA
[2]Institut de Recherche en Sciences de la Santé/Centre Muraz, Bobo-Dioulasso, Burkina Faso
[3]MIVEGEC, IRD, CNRS, University of Montpellier, Montpellier, France
[4]Howard Hughes Medical Institute, Chevy Chase, MD, USA
*Correspondence: fcatter@hsph.harvard.edu, thierry.lefevre@ird.fr

Abstract (<300 words):

Anopheles gambiae and its sibling species *Anopheles coluzzii* are the most efficient vectors of the malaria parasite *Plasmodium falciparum*. When females of these species feed on an infected human host, oogenesis and parasite development proceed concurrently, but interactions between these processes are not fully understood. Using multiple natural *P. falciparum* isolates from Burkina Faso, we show that in both vectors, impairing steroid hormone signaling to disrupt oogenesis leads to accelerated oocyst growth and in a manner that appears to depend on both parasite and mosquito genotype. Consistently, we find that egg numbers are negatively linked to oocyst size, a metric for the rate of oocyst development. Oocyst growth rates are also strongly accelerated in females that are in a pre-gravid state, i.e. that fail to develop eggs after an initial blood meal. Overall, these findings advance our understanding of mosquito-parasite interactions that influence *P. falciparum* development in malaria-endemic regions.

Author Summary (150-200 words):

Malaria, an infectious disease caused by *Plasmodium* parasites, continues to affect millions of people each year. These parasites are transmitted by *Anopheles* mosquitoes during blood feeding, which is required by females to produce eggs. After they are ingested by the female, parasites start developing in the midgut at the same time as the mosquito initiates oogenesis, the process of egg development. Here, we investigated how the concomitant processes of egg and parasite development interact in field-derived *Anopheles* and *Plasmodium* isolates. We found that in these naturally occurring pairs, oogenesis is negatively linked to parasite development, such that mosquitoes producing

fewer eggs harbor faster growing oocysts. Furthermore, when we disrupt signally pathways that drive oogenesis, oocyst growth is accelerated in a manner that appears to depend on both mosquito and parasite genotype. Additionally, when field mosquitoes fail to develop eggs, they are likely to have faster growing oocysts. The interaction between key mosquito and parasite developmental processes revealed here underscores how intimately linked *Plasmodium* are to mosquito physiology and can help inform the development of future vector control tools that are both safe and effective.

Introduction

Plasmodium parasites, the causative agents of malaria, are transmitted by female mosquitoes from the *Anopheles* genus. Like all anautogenous mosquitoes, female *Anopheles* require a blood meal to produce eggs, and parasites take advantage of this requirement to enable their transmission between human hosts. Blood feeding triggers a vast metabolic and transcriptional program, largely regulated by the insect steroid hormone 20-hydroxyecdysone (20E), which ultimately results in the production of an egg batch (1, 2, 3). Concurrently, ingested parasites begin their development (sporogony) in the mosquito gut when male and female gametes fuse to form a zygote, which then rapidly transforms into a motile ookinete that breaks across the midgut epithelium and settles beneath the basal lamina to form a sessile oocyst. Oocysts subsequently grow over several days producing thousands of sporozoites, which are eventually released into the mosquito hemolymph and invade the salivary glands, from where they can be transmitted to the next human host when the female blood feeds again.

One critical aspect of *Plasmodium* infection in the mosquito is the rate at which oocysts develop, as this ultimately determines the extrinsic incubation period (EIP) (4), or the length of time it takes parasites to complete sporogonic development and reach transmission stages. The EIP is a key determinant of malaria transmission due to the lengthy parasite developmental cycle. Indeed, *Plasmodium falciparum*, the deadliest form of human malaria, requires a minimum of 9 days for development (5, 6, 7, 8), mostly due to the extensive time needed for oocyst growth. This time largely corresponds to the rather limited lifespan of female mosquitoes in the field, which is variable (9) but estimated to last for 2-3 weeks (10, 11, 12)).

Factors known to affect the length of *P. falciparum* sporogony are temperature (13, 14, 15, 16), adult diet (17), larval nutrition (18), and multiple blood meals (7, 19, 20) (21, 22). Additionally, we have demonstrated that the rate of *P. falciparum* development in *Anopheles gambiae*—a major malaria vector across much of sub-Saharan Africa—is also linked to the hormonally-regulated processes leading to oogenesis. Impairing the mosquito's ability to produce eggs through several methods, including disrupting the function of the steroid hormone 20E via silencing its heterodimeric nuclear receptor *Ecdysone Receptor* (*EcR*)/*Ultraspiracle*, resulted in faster oocyst development. This in turn resulted in sporozoites reaching the salivary glands sooner while, crucially, still maintaining full infectivity to human hepatocytes (23). Moreover, we determined that egg numbers are negatively correlated to *P. falciparum* growth rates, a finding implying that mosquitoes with reduced reproductive output can transmit parasites sooner, thereby providing parasites with a significant advantage given the short mosquito life span.

Understanding whether this interaction is relevant in malaria-endemic regions where different *P. falciparum* genotypes and multiple *Anopheles* species coexist is essential to reveal the complex dynamics of this vector-parasite interplay.

Here, we examine interactions between oogenesis and sporogony in natural settings in Burkina Faso using multiple *P. falciparum* isolates collected from gametocyte donors and field-derived colonies of two *Anopheles* species. We determine that silencing the 20E nuclear receptor *EcR* in *An. gambiae* and its sibling species *Anopheles coluzzii* leads to faster parasite development. In agreement with these findings, in both species, we find that oocyst growth rates are negatively linked to oogenesis such that females naturally producing fewer eggs harbor faster developing oocysts. We observe variability in the oocyst growth response to *EcR* silencing across different *P. falciparum* isolates and between the two *Anopheles* species, suggesting that both parasite and mosquito genotype are determinants of growth. Additionally, parasite growth rates are significantly accelerated in field-collected *An. coluzzii* females that are in a pre-gravid state (i.e. unable to develop eggs after an initial blood meal), a very common phenomenon in natural mosquito populations (24, 25). Overall, our data across diverse *Anopheles-Plasmodium* combinations suggest that parasite development is favored in females that have reduced or impaired oogenesis, expanding our understanding of factors affecting sporogony in the field. These data also have implications for malaria control strategies that aim to interfere with mosquito reproduction.

Results

Impairing 20E signaling leads to accelerated *P. falciparum* oocyst growth in both *An. coluzzii* and *An. gambiae*

To investigate if *P. falciparum* development is affected by *Anopheles* oogenesis in field settings, we performed infection experiments in Burkina Faso with naturally circulating *P. falciparum* isolates collected from six gametocyte carriers, here denoted as patient 1-6 (p1-p6) (26). We used colonies of *An. coluzzii* and *An. gambiae* that are frequently replenished from local wild populations. In both species, we impaired 20E signaling via RNAi silencing of the ecdysone nuclear receptor (ds*EcR*), or *green fluorescent protein* (ds*GFP*) as a control. Three *P. falciparum* isolates were used to infect *An. coluzzii* (p1-p3) (**Fig 1A**), and five were used to infect *An. gambiae* (p1, p3-p6) (**Fig 1B**).

We first assessed egg development in these infected females and determined that while eggs were only marginally affected by ds*EcR* treatment in *An. coluzzii* (**Fig 1C, S1A Fig**), they were significantly decreased in *An. gambiae* following ds*EcR* injections (**Fig 1D, S1B Fig**). We next counted oocyst numbers and found that *EcR* silencing had no effect on the prevalence or intensity of infection in either species (**Fig 1E-F, S2A-B Fig**), partially in contrast to our laboratory observations showing a reduction in oocyst numbers for *An. gambiae* in these conditions (23). We went on to determine parasite growth rates by measuring the cross-sectional area of oocysts at 8 days post-infected blood meal (pIBM)(23) and found a striking increase following ds*EcR* injections in both *Anopheles* species. Specifically, in EcR-depleted females *P. falciparum* oocysts were 55% larger in *An. coluzzii* and 78% larger in *An. gambiae* as compared to their respective ds*GFP* controls (**Fig 2A-C**).

Our studies using cultured NF54 parasites had shown that larger oocysts are indicative of faster parasite development (7, 23). Although we did not measure EIP directly in this study, we found that ds*EcR An. gambiae* females had a 5.5-fold increase in the number of sporozoites in their salivary glands 12 days after infection relative to controls (**Fig 2D**). Notably, in our experimental settings (23), 12 days pIBM is a time point when sporozoites have started but not yet completed invasion of the salivary glands, so it is likely that this increase in sporozoites reflects faster parasite development. This trend was also observed across individual infections, even though they lacked power when analyzed independently (**S3 Fig**). Sporozoite prevalence also appeared higher in ds*EcR* females at 12 days pIBM, but the effect was not statistically significant (pie charts in **Fig 2D, S3 Fig**).

To determine a possible mechanism behind accelerated growth, we measured expression levels of the lipid transporter *lipophorin* (*Lp*), previously implicated in the faster oocyst growth rates observed in laboratory depletions of EcR (23). We detected elevated *Lp* expression in ds*EcR* females of *An. coluzzii* and *An. gambiae* (**Fig 2E**), which could allow greater provisioning of lipids to the developing oocysts (23). Combined, these data show that when 20E signaling is disrupted, growth of *P. falciparum* field isolates is accelerated, and this effect may be mediated by Lp-transported lipids, consistent with previous findings (23).

Parasite growth is negatively linked to egg development via 20E signaling

We next determined the relationship between oocyst size and egg numbers within our two *Anopheles* species. To do this, we used a Generalized Linear Mixed-Effects Model (GLMM) (27, 28) to analyze paired egg-oocyst data collected from all *P. falciparum*-infected females (**S1 Table, S2 Table**). In *An. coluzzii,* there was a significant two-way interaction between egg number and ds*EcR* treatment (**S1 Table**), suggesting that the effect of egg development on oocyst size differs between control and ds*EcR* females. Consistent with this, when analyzed independently, while *An. coluzzii* controls showed a significant negative association between egg numbers and oocyst size, in *EcR*-silenced females this association was lost (**Fig 3A**). These findings mean that in *An. coluzzii*, oocysts tend to be larger in females that naturally produce fewer eggs, and conversely, females that develop more eggs are likely to have smaller oocysts, but impairing 20E-signaling breaks this link between egg and oocyst development.

Instead in *An. gambiae* we found a complex three-way interaction between egg number, ds*EcR* treatment, and oocyst number (**S2 Table**), suggesting that both 20E signaling and oocyst density regulate the egg-oocyst relationship. When analyzed independently, both control and *EcR*-silenced females have a general negative association between egg numbers and oocyst size (**Fig 3B**), and this association is strongest at low (<10) oocyst densities in controls and at high (>30) oocyst densities in ds*EcR* females (**Fig 3C**). As with *An. coluzzii*, these findings demonstrate that oocyst growth is inversely linked to a female's investment in reproduction, likely because resources that are not utilized for oogenesis become available to parasites. All together these data reveal that egg development is an important factor affecting oocyst growth in two *Anopheles* species, but

also, that signaling by the steroid hormone 20E may differently regulate the egg-oocyst interaction in these mosquito genetic backgrounds.

Parasite isolates vary in their growth response to impaired 20E signaling

As malaria is highly prevalent in the villages surrounding Bobo-Dioulasso (29, 30), we reasoned that individual patients may have polyclonal infections comprised of multiple *P. falciparum* genotypes. Indeed, we found that most of the isolates used in our infections contain two or more distinct parasite genotypes, as determined by analyzing the *merozoite surface protein 1* (*MSP1*) (31) (**Table 1, S3 Table**). When we assessed parasite growth in the individual p1-p6 infections (**Fig 4A, B**), we found that oocyst size does not vary between parasite isolates in ds*GFP* controls (*An. coluzzii* ds*GFP*: GLMM, LRT $X^2{}_2 = 1.52$, $p = 0.466$; *An. gambiae* ds*GFP*: GLMM, LRT $X^2{}_4 = 3.47$, $p = 0.483$). On the other hand, in ds*EcR* females, we detected significant differences between isolates (*An. coluzzii* ds*EcR*: GLMM, LRT $X^2{}_1 = 6.3$, $p = 0.012$; *An. gambiae* ds*EcR*: GLMM, LRT $X^2{}_1 = 4.67$, $p = 0.031$). Despite some inevitable variability due to using different mosquito batches, all isolates increased in size in ds*EcR* females when compared to controls, with the notable exception of p3 in *An. coluzzii* (**Fig 4A**) and p6 in *An. gambiae* (**Fig 4B**). It is important to note that p6 parasites infected the same batch of *An. gambiae* mosquitoes as p5, which instead showed the characteristic size increase in response to *EcR* silencing (**Fig 4B**). As p5 and p6 differ in both the number and combination of genotypes they contain (**Table 1, S3 Table**), these observations suggest that parasite genetic factors may influence the interactions between mosquito oogenesis and *P. falciparum* growth rates. Interestingly, we observed that p3 parasites did not respond to *EcR* silencing in *An.*

coluzzii (**Fig 4A**), yet they increased in size in *An. gambiae* (**Fig 4B**), emphasizing that the same parasite isolate can behave differently in different *Anopheles* species. The only other parasite isolate tested in both species, p1, responded robustly to *EcR* silencing in both vectors. With the caveat that we could perform only two direct comparisons between *An. gambiae* and *An. coluzzii*, these data suggest that the mosquito genotype may also play a role in determining parasite growth rates. Further supporting this idea, in controls infected with p1 or p3 parasites, we see that oocyst growth is significantly different between our two mosquito species, whereby p1 and p3 oocysts grow larger in *An. coluzzii* than in *An. gambiae* (GLMM, LRT X^2_1 = 4.03, p = 0.045, **Fig 4C**).

Table 1: Gametocytemia and COI for *P. falciparum* field isolates

Parasite Isolate	Gams/µL	COI
**p1**	128	3
**p2**	208	2
**p3**	120	1-3
**p4**	56	1-3
**p5**	72	3
**p6**	64	2

P. falciparum parasites were collected from six different gametocyte carriers (p1-p6) and used for infections in *An. coluzzii* and *An. gambiae* females. Prior to infection, a blood smear was used to count the number of gametocytes present in each sample. Gametocytes were counted per 1,000 leukocytes and then, using an estimated conversion factor of 8,000 leukocytes per uL of blood, converted to gametocytes per µL of blood (Gams/µL). *MSP1*-typing was performed on dried blood spots or recently thawed frozen blood samples and used to determine the (minimum) number of unique parasite genotypes, or complexity of infection (COI), present in each isolate.

Oocyst growth is accelerated in pre-gravid mosquitoes

Given the observed negative association between mosquito oogenesis and parasite growth rates (**Fig 3**), we next set out to investigate oocyst development in pre-gravid mosquitoes (females that fail to develop eggs after taking an initial blood meal), using field-derived *Anopheles* because this phenomenon is highly common in wild populations and considerably less so in laboratory colonies (24, 25, 32). To this aim, we collected *An. coluzzii* larvae from natural breeding sites and infected emerged adult females with the same six gametocyte carriers as above (p1-p6). Out of the 323 mosquitoes analyzed in these experiments, the vast majority (86%, N = 277) failed to develop eggs despite taking a blood meal (**S4A Fig**). This effect could be driven by the fact that field mosquitoes are not well-adapted to membrane feeding and may take smaller blood meals, but additionally, it may also suggest that the pre-gravid rate is high in this population as previously observed (24, 33).

Altogether, the field mosquitoes averaged 72% infection prevalence with a mean of 11 oocysts per blood feeding event (**S4B Fig**). The pre-gravid females were slightly less likely to become infected than gravid females (pie charts in **Fig 4D**), yet among infected individuals, there was no difference in oocyst intensity (**Fig 4D**). We next determined oocyst size, for practical reasons at 7 days pIBM, one day earlier than in previous experiments. Strikingly, oocysts were significantly larger in pre-gravid females as compared to females that produced an egg batch, with a 48% size increase (**Fig 4E**)(**S4 Table**). Although limited in number, the same trend was observed for the colony mosquitoes (**S4C Fig**). Therefore, pre-gravid status in *An. coluzzii* has two contrasting

effects on parasite infection within the mosquito – it reduces infection prevalence but also strikingly promotes oocyst growth, with probable effects on the EIP.

Discussion

In this study, we investigated the interplay between mosquito oogenesis and parasite development using multiple *P. falciparum* field isolates and two different *Anopheles* species, including field collected mosquitoes. We found that oocyst size, a good metric for oocyst growth rates (7, 23), is negatively linked to oogenesis in *An. coluzzii* and *An. gambiae*, such that disruption of 20E signaling resulted in accelerated oocyst growth in both species (**Fig 2A-C**) and across several parasite isolates (**Fig 4A-B**). In *An. gambiae*, ds*EcR* treatment also resulted in a greater number of salivary gland sporozoites at the relatively early time point of 12 days piBM (**Fig 2D, S3 Figure**). While additional studies assessing sporozoite load across time are still needed to precisely quantify the effects on the EIP, these data confirm that impairing the processes regulating mosquito oogenesis can result in earlier arrival of sporozoites to the salivary glands. Given the lengthy sporogonic cycle of *P. falciparum* (minimum 9 days (5, 6, 7)) coupled with the relatively limited lifespan of mosquitoes in the field (~2-3 weeks (10, 11, 12)), parasites that develop faster will have a greater chance of being transmitted (7). Although here we could not perform hepatocyte infections due to restrictions in the experimental set-up, our previous laboratory studies showed that faster developing parasites are just as transmissible to human hepatocytes as slower developing parasites (23).

Consistent with accelerated parasite growth in ds*EcR* females, we also found that in both *An. coluzzii* and *An. gambiae* controls, egg development is negatively linked to oocyst size (**Fig 3A-B**), confirming that *P. falciparum* growth is influenced by female reproductive processes (23). In *An. gambiae*, the effect of oocyst density on this interaction suggests that at low density infections, any change in the pathways leading to egg development results in a greater per-oocyst change in available resources, therefore affecting oocyst growth more profoundly than at high density infections (**Fig 3C**). Disruption of 20E-signaling perturbs the effect of parasite density on these egg-oocyst size interactions, but in contrast to *An. coluzzii*, the negative link between egg numbers and oocyst size remains intact (**Fig 3A, B**). Overall, these results demonstrate that there is a strong, conserved interaction between *Anopheles* oogenesis and oocyst growth, but also that 20E signaling regulates this interaction differentially, even in closely related species, indicating that *P. falciparum* interacts uniquely with the post-blood meal environments of these two anophelines. Previously, we demonstrated that the negative association between egg development and oocyst growth is dependent on midgut lipids that the parasite may access via Lipophorin (23). Given that we observed elevated *Lp* expression in the ds*EcR*-treated females (**Fig 2E**), it is likely that resource allocation and lipids/lipid transporters also play a role in the oocyst growth phenotypes we observed here in the field, although this remains to be tested directly.

Further supporting the link between oogenesis and parasite growth, in infections with field-collected *An. coluzzii*, pre-gravid females that failed to develop eggs had significantly larger oocysts compared to females that produced an egg batch (**Fig 4E**). On the other

hand, pre-gravid females were also less likely to become infected initially (pie charts in **Fig 4D**), as also seen previously (17), perhaps because these females take smaller blood meals and therefore are less exposed to infectious parasites. These contrasting effects of the pre-gravid status on two major determinants of vectorial capacity (vector competence and speed of parasite development) make it difficult to conclude how pre-gravid mosquitoes may contribute to malaria transmission dynamics, and models of transmission would be needed to assess this fully. Despite this limitation, given that the pre-gravid condition is quite common and pre-gravid rates over 60% have previously been reported for certain *Anopheles* field populations (24, 33), possibly due to low teneral reserves (32, 33, 34), our observations that pre-gravid females favor oocyst growth may suggest that the EIP of parasites in field settings is actually shorter than that typically observed with lab-adapted colony mosquitoes. Furthermore, the effect of pre-gravid status on oocyst growth rates could be even further exacerbated by these females needing to seek an additional blood meal to complete production of their first egg batch, since it has been shown that a second blood meal significantly accelerates sporozoite formation (7).

In our experiments, oocyst intensity and prevalence were not affected by *EcR* silencing in either *An. coluzzii* or *An. gambiae* females (**Fig 1E-F**). This is partly in contrast to our previous study where we found a reduction in parasite numbers (but not prevalence) during the ookinete-oocyst transition. Considering that parasite numbers are roughly 4 times lower here than those achieved in laboratory conditions (23), it is possible that the effects of 20E signaling on parasite survival are dependent on the intensity of infection.

Given that oocyst density was also a significant factor affecting egg-oocyst size relationships in *An. gambiae* (**Fig 3C**), it would be worthwhile to further explore how infection load may influence these multiple interactions between parasites and oogenetic processes. Interestingly, ds*EcR* treatment did not significantly reduce egg numbers in *An. coluzzii*, while it did in *An. gambiae* (**Fig 1C-D**). Due to the low levels of *EcR* expression in females prior to blood feeding, knockdown efficiency was confirmed via increased expression of the lipid transporter *Lp* (one of the genes that is strongly induced by *EcR* silencing (23))(**Fig 2E**), suggesting that silencing was comparable in these species. A possible explanation for these results is that 20E regulation of post-blood feeding processes is slightly different in these two species, as also indicated by the differential effects of ds*EcR* treatment on the egg-oocyst size interaction in *An. coluzzii* compared to *An. gambiae* (**Fig 3A-B**). Regardless, disrupting 20E signaling resulted in significantly larger oocysts in both anophelines (**Fig 2A-C**), demonstrating that 20E regulated pathways have a conserved influence on parasite growth.

While most parasite isolates responded to impaired 20E signaling by accelerating development, the comparison between p5 and p6 in the same batch of *An. gambiae* revealed that p6 parasites did not increase in size (**Fig 4B**). Furthermore, p3 oocysts did not respond to *EcR* silencing in *An. coluzzii* (**Fig 4A**), yet they had a large growth response in *An. gambiae* (**Fig 4B**). Taken together, these results suggest that mosquito-parasite genotype-by-genotype interactions may regulate sporogonic development, as postulated for dengue virus EIP in *Aedes* (35). This conclusion is further supported by our GLMM analyses, which revealed that mosquito genotype (ds*GFP An. gambiae* vs ds*GFP*

An. coluzzii) (**Fig 4C**) and parasite isolate (p1-p6 in ds*EcR* females) (**Fig 4A-B**) can significantly influence oocyst growth rates. Considering that high levels of genetic diversity exist in parasite populations in regions with high malaria transmission (36, 37, 38) such as Burkina Faso (39), there is likely natural variability in parasite genes regulating development (e.g. genes involved in metabolism, or nutrient transporters). It is plausible that these differences may be caused by the parasite's need to adapt to and be transmitted by different sympatric *Anopheles* species populating the same geographical area (40, 41, 42), thus maintaining polymorphisms in genes affecting oocyst growth rates.

Interestingly, we found that oocyst growth significantly varied across parasite isolates in *EcR*-silenced mosquitoes (*An. gambiae* and *An. coluzzii*) (**Fig 4A-B**, ds*EcR*), yet we failed to detect any isolate-driven effect on oocyst growth in ds*GFP* controls (**Fig 4A-B,** Cntrl) or wild VK5 *An. coluzzii* **(Fig S4D**). This may be because the effects of parasite genotype on oocyst growth are only detectable through our method of oocyst measurement in conditions where a great surplus of nutrients are accessible to the parasite throughout development, as when 20E signaling is impaired (23). Isolate-specific differences in parasite growth may emerge more clearly following additional blood meals, when growth rates are profoundly accelerated (7, 19, 20). Providing a second blood meal to these mosquitoes would be most representative of field conditions, as pre-gravid mosquitoes will require a second feed into order to produce eggs.

Altogether, this study expands our knowledge of naturally occurring mosquito-parasite interactions that influence the rate of sporogony in the field. These findings are important

for understanding the dynamics of *P. falciparum* transmission in sub-Saharan Africa, and for informing the development of future vector control strategies that seek to interfere with mosquito reproduction. Indeed, our data emphasize that mosquito species may differ in their interactions with parasite isolates. Manipulations to fundamental processes like mosquito reproduction will need to be evaluated for their effects within the specific contexts in which control strategies are implemented and cannot necessarily be extrapolated from laboratory experiments that use single combinations of lab-adapted mosquito and parasite strains.

Materials and Methods

Ethical Approval

Ethical approval was obtained for all procedures involving human subjects from institutional review boards in both the United States and Burkina Faso. Approval through Harvard University was obtained for project # IRB17-0609. This is in agreement with project #18-2018/CEIRES, which was approved by the Institutional Ethics Committee for Research in Health Sciences at the Ministry of Scientific Research, Burkina Faso. Informed written consent was obtained from a parent or guardian of all volunteers prior to participation in the study.

Mosquito Rearing

Experiments were performed with either *An. gambiae* or *An. coluzzii* mosquitoes from independently maintained colonies that were both established in 2008 from gravid females collected in south-western Burkina Faso: Soumousso for *An. gambiae* and Kou Valley for *An. coluzzii* (43). These colonies are frequently replenished with the F1 offspring of wild-caught, gravid females and species identification is performed using a SINE200 diagnostic PCR (44). For experiments with field-collected *An. coluzzii,* larvae were collected from breeding sites in VK5, Kou Valley, Burkina Faso and then reared in the laboratory until pupal stages.

Pupae were sex sorted and then allowed to emerge into cages, where adults were maintained on a 10% glucose solution provided *ad libitum* and under standard rearing

conditions (27 °C, 70-80% humidity, 12h day/night cycle). Female mosquitoes were maintained as virgins throughout adulthood. Prior to blood feeding, mosquitoes were mouth aspirated from cages into large cups sealed with netting and sugar-starved for ~24hr. After blood feeding, engorged mosquitoes were returned to cages and again maintained as before on 10% glucose solution under standard conditions. Before any dissections, mosquitoes were cold-anesthetized and decapitated.

Gene knockdown using dsRNA

dsRNA production: *EcR* (*AGAP029539*; 435 bp) and eGFP (495 bp) fragments were amplified by PCR from the plasmids pCR2.1-EcR and pCR2.1-eGFP (confirmed by DNA sequencing) using universal primers against the pCR2.1 backbone and adding the T7 promoter, as described in (23, 45). Primer sequences were (with T7 promoter in lowercase): pCR2.1-T7F: 5'–taatacgactcactatagggCCGCCAGTGTGCTGGAA–3'; pCR2.1-T7R: 5'–taatacgactcactatagggGGATATCTGCAGAATTCGCCC–3'. After amplification, PCR products were transcribed into dsRNA using the Megascript T7 transcription kit (Thermo Fisher Scientific) and then purified, as described previously (46). dsRNA was aliquoted at a concentration of 10 µg/µl and frozen for transport on dry-ice from Boston, USA to Bobo-Dioulasso, Burkina Faso. Prior to experimental use, gel electrophoresis was used to confirm dsRNA quantity and integrity.

dsRNA injections: Adult virgin females <24h post-eclosion were anesthetized on ice and then injected in the thorax with 69 nL of dsRNA (ds*GFP* or ds*EcR*) at a

concentration of 10 µg/µl using a Nanoject II (Drummond). Immediately following injection, females were transferred to a large cup for recovery before being moved to a new cage where they were maintained under standard rearing conditions as described above; only females surviving injections (>90%) were used in experiments. 3–4 days post-injection, females were provided with a *P. falciparum*-infected blood meal.

Gene expression analysis: *Sample collection:* On the same day that mosquitoes were provided a *P. falciparum*-infected blood meal (3–4 days post-injection), non-blood fed females were collected for gene expression analysis. Females were cold-anesthetized, de-capitated, and then the rest of body samples were stored in 500 µl of RNAlater (Invitrogen). 2 pools of 5 mosquitoes were collected from both ds*GFP* and ds*EcR* groups, with collections done for each batch of mosquitoes that was infected (3 batches of *An. gambiae* and 2 batches of *An. coluzzii* (**Fig 1A, B**)). Samples in RNAlater were kept for ~1 day at room temperature or 4°C, before being frozen at -20°C. During transport back to Boston, USA from Bobo-Dioulasso, Burkina Faso, the samples were at room temperature for ~3 days, before again returning to -20°C for long-term storage.

RNA extraction & cDNA synthesis: Using sterile forceps, mosquito samples were removed from the collection tubes containing RNAlater, blotted on a Kimwipe, and then transferred to a fresh set of tubes containing 400 µl of TRI Reagent (Thermo Fisher Scientific). A bead-beater was used to homogenize the samples using two 2-mm beads per tube and beating at 2400 rpm for 2 rounds of 90 seconds each (chilling samples at -20°C for 5 min between rounds of homogenization). Following homogenization, RNA extraction was performed according to TRI Reagent manufacturer instructions. After

extraction, Turbo DNAase (Thermo Fisher Scientific) was used to DNAse treat the samples. NanoDrop Spectrophotometer 2000c (Thermo Fisher Scientific) was used both before and after DNAase treatment to assess the quality and quantity of RNA. About 3 µg of RNA was used for cDNA synthesis, performed as described in (47).

qRT-PCR: EcR expression is very low in non-blood fed females and unreliable for direct assessment of gene knockdown efficiency; therefore, transcript levels of the mosquito lipid transporter *Lipophorin* (*Lp*) were used as a reporter for *EcR* knockdown, because *Lp* is known to be strongly induced by *EcR* silencing independent of blood feeding (23) (**Fig 2E**). To this end, quantitative real-time PCR (qRT-PCR) was performed on a QuantStudio 6 Pro thermocycler (Thermo Fisher Scientific) in 15 µl reactions containing 1X PowerUp SYBR Green Master Mix (Thermo Fisher Scientific), primer dilutions, and 5 µl of sample cDNA diluted 1:10. Primers (Integrated DNA Technologies) targeting *Lipophorin* (*Lp*) (*AGAP001826*) or the ribosomal gene *RpL19* (*AGAP004422*), used as a housekeeping control, were previously published (23). Relative gene expression was determined using the delta-delta-Ct method, normalizing *Lp* expression against *RpL19*, and ds*EcR* samples against ds*GFP*.

Infections with *P. falciparum* field isolates

Identification of gametocyte carriers: *P. falciparum* gametocyte carriers were identified in the villages surrounding Bobo-Dioulasso, as described in (48, 49). Briefly, children 5–13 years old were surveyed for the presence of *P. falciparum* parasites by fingerprick blood smears, which were analyzed by Giemsa staining and light microscopy. Subjects were asked if they would participate in the study if they were found

to have a gametocytemia between 5–40 gametocytes per 1000 leukocytes (or 40–320 gametocytes/µl blood, based on an estimated conversion factor of 8000 leukocytes/µl blood). All persons found to be infected with malaria from this screen were offered treatment regardless of enrollment in the study.

Membrane feeding: If parental consent was granted, gametocyte carriers were brought to the lab where < 6 ml of venous blood was drawn by a trained technician. Blood was spun down at 1800 rpm for 5 min at 37°C to separate the red blood cells from the serum, and then the sample serum was removed and replaced with purchased serum from persons naïve to malaria (48, 50). After resuspension, the blood was introduced into 20–30 glass membrane feeders heated to 37°C with a Haake water pump. Mosquitoes contained in large cups were allowed to feed through a parafilm membrane sealing the bottom of the glass feeders for 60–120 mins (49, 50, 51). Remaining sample blood was maintained at 37°C and supplemented into the feeders as needed. Once feeding time was complete, any unfed or partially-fed females were mouth aspirated and removed from the experiment.

Assessing fecundity and pre-gravid status

Since females were maintained as virgin throughout experimentation, oviposition did not occur following blood feeding and fecundity/pre-gravid status could be determined at the same time that *P. falciparum* midgut infection was assessed 7–8 days pIBM. At this time, ovaries were dissected and developed eggs were counted (dsRNA-injected *An. gambiae*, *An. coluzzii*) or females were scored as gravid/pre-gravid (VK5 *An.* coluzzii). Assessing

egg development concurrently with midgut dissections allowed for paired egg-oocyst data collection.

Assessing *P. falciparum* infection in the midgut

7 days (VK5 *An.* coluzzii) or 8 days (dsRNA-injected *An. gambiae*, *An. coluzzii*) post-infected blood meal, female midguts were dissected and stained in 1% mercurochrome. Light microscopy was then used to count the number of *P. falciparum* oocysts present in the midgut, determining the prevalence and intensity of infection. Images of the infected midguts were also obtained, and from these images oocyst size was determined by manually measuring the cross-sectional area of all intact oocysts using FIJI software (52). The average size of all oocysts present in a single midgut was calculated for each female and used for subsequent analysis. Any blurry midgut images, for which oocyst measurements could not be reliably determined, were excluded from our analysis.

Assessing sporozoites in the salivary glands

Individual female salivary glands were dissected into 1X phosphate-buffered saline (PBS) at 12 days pIBM. The salivary glands were hand homogenized using a plastic pestle and spun at 8000 *g* for 10 min. The supernatant was discarded and the remaining pellet (containing sporozoites) was resuspended in PBS to a known volume. Phase-contrast microscopy was used to count sporozoites present in 0.1 µl using disposable hemocytometers. These counts were then used to calculate the total number of sporozoites present in the salivary glands of each mosquito. The lower limit of detection for an individual female was ~120 sporozoites.

Genotype analysis of *P. falciparum* field isolates

Collection of blood spots and frozen blood samples: Immediately following collection from gametocyte carriers, a small volume of blood was spotted onto Whatman paper to generate dried blood spots. Additionally, a small volume of the blood (~1 ml) was added to Glycerolyte 57 (Baxter Fenwal 4A77831) at a 1:1 ratio and aliquoted into Nunc cryotubes. The samples were kept at -80°C until they were shipped on dry-ice back to Boston, USA and transferred to liquid nitrogen for long-term storage.

Parasite DNA extraction & confirmation of *P. falciparum* infection: DNA was extracted from dried blood spots or recently thawed frozen blood samples (in the case of p5 and p6) using a DNeasy Blood and Tissue kit (Qiagen), involving lysis at 56°C, column purification, and elution. Extracted DNA was then used for a nested PCR of 18S rDNA according to Snounou *et al* (53) to confirm that only *P. falciparum* parasites, and no other human *Plasmodium* species, were present in the samples. Primers for the first PCR reaction were:

rPLU-F: 5'–TTAAAATTGTTGCAGTTAAAACG–3';

rPLU-R: 5'–CCTGTTGTTGCCTTAAACTTC–3'.

The subsequent, nested PCR reactions used primers specific for *P. falciparum*, *P. malariae,* or *P. ovale* with the following sequences, respectively:

rFAL-F: 5'–TTAAACTGGTTTGGGAAAACCAAATATATT–3';

rFAL-R: 5'–ACACAATGAACTCAATCATGACTACCCGTC–3'.

rMAL-F: 5'–ATAACATAGTTGTACGTTAAGAATAACCGC–3';

rMAL-R: 5’–AAAATTCCCATGCATAAAAAATTATACAAA–3’.

rOVA-F: 5’– ATCTCTTTTGCTATTTTTTAGTATTGGAGA–3’;

rOVA-R: 5’– GGAAAAGGACACATTAATTGTATCCTAGTG–3’.

Gel electrophoresis was used to evaluable all PCR products and confirm that only *P. falciparum* parasites were present in our samples (p1-p6).

***MSP1*-typing and COI determination:** Extracted sample DNA was used to determine the *MSP1* genotype(s) present in each sample by nested PCR as described by (31). This nested PCR approach was used to identify the allelic type of 4 variable regions on the *MSP1* gene termed block 2, 4A, 4P, and 6. Each block can either have a MAD20 or K1 allelic family type, with the exception of block 2, which can alternatively have an RO33 type. The first PCR reaction was used to type blocks 2 and 6. To do this, the following primers (3 forward; 2 reverse) were used in six unique combinations for each parasite isolate:

M2F: 5’– GGTTCAGGTAATTCAAGACGTAC–3’

K2F: 5’– TCTTAAATGAAGAAGAAATTACTACAAA–3’

R2F: 5’– TAAAGGATGGAGCAAATACTCAAGT–3’

M6R: 5’– ATTTGAACAGATTTCGTAGGATCTTG–3’

K6R: 5’– GATATTCATTGGGTTCAGTTGATTC–3’

PCR products were then run on a gel in order to identify blocks 2 and 6 as: M--M, M--K, K--M, K--K, R--M, or R--K. PCR products from the first reaction were then used in subsequent nested PCRs where blocks 4A and 4P were typed. This second round of PCRs was done using the following primers (2 forward; 2 reverse) in four unique combinations:

M4AF: 5'– TTGAAGATATAGATAAAATTAAAACAGATG–3'

K4AF: 5'– AATGAAATTAAAAATCCCCCACCGG–3'

M4PR: 5'– TCGACTTCTTTTTTCTTATTCTCAG–3'

K4PR: 5'– TCCTCGATTTTTTTGTTCTTATCAAG–3'

The resulting PCR products were run on a gel, identifying blocks 4A and 4P as: -MM-, -MK-, -KM-, or -KK-. Combining the typing results from all four blocks (*i.e.* MMMM, KMMK, RMKK, etc), allows for the identification of up to 24 distinct *MSP1* genotypes. These nested PCR reactions were performed on blood sample DNA (p1-p6) to determine which *MSP1* genotypes were present in each isolate (**S1 Table**). The number of unique *MSP1* genotypes identified in each sample was then used to determine the sample's (minimum) complexity of infection (COI) (**Table 1**). NF54 cultured parasites were used a control for these experiments, as they are clonal and known to contain only a single parasite genotype.

Statistical Analyses

GraphPad Prism 8, JMP Pro 14, and R were used to analyze the data with a significance cut-off of p = 0.05. Oocyst size data was analyzed using Generalized Linear Mixed Effects Models (GLMM) and Likelihood Ratio Tests (LRT), allowing for nesting of individual oocyst measurements by mosquito, and assessment of both fixed and random effects. Other continuous data (egg, oocyst, and sporozoite number) was first analyzed for normality (D'Agostino/Pearson and KS normality test) and transformed to generate normally distributed data when possible. If normally distributed, data was then analyzed by either unpaired t-test or one-way ANOVA (Welch's correction when appropriate). Non-

normally distributed data was analyzed by either Mann-Whitney or Kruskal-Wallis. Prevalence data (for oocysts and sporozoites) was analyzed using Fisher's Exact test. Specific statistical tests and p values are indicated in the text, figures, and figure legends. All graphs display mean values with standard error of the mean (SEM).

Acknowledgements

We wish to thank all the students and technicians at the IRSS/IRD who provided valuable assistance for the experiments of this study. We especially wish to thank Hélène Somé, Baudoin Dabiré and Fulgence Da for mosquito rearing, Charles Nignan for coordinating VK5 larval collections, and Souleymane Tamboula for assistance removing unfed females following infectious feeds. We also wish to thank Perrine Marcenac for helpful discussions of the data and Manuela Bernardi for graphical artwork. Finally, we'd like to thank the local villagers and study participants.

Funding Statement

This work was funded by the National Institutes of Health (NIH) (R01 AI124165, R01 AI104956) and by a Faculty Research Scholar Award by the Howard Hughes Medical Institute (HHMI) and the Bill & Melinda Gates Foundation (BMGF) (Grant ID: OPP1158190) to FC. FC is a HHMI Investigator. The funders had no role in the study design, data collection, data analysis, data interpretation, decision to publish or preparation of the manuscript. The findings and conclusions herein do not necessarily reflect positions or policies of the NIH, HHMI or BMGF.

Declaration of Interests

The authors declare no competing interests.

Figure Legends

Fig 1: *An. coluzzii* and *An. gambiae* females were infected with *P. falciparum* field isolates following *EcR*-silencing. (A-B) *An. coluzzii* and *An. gambiae* females were injected with either ds*GFP* (Cntrl) or ds*EcR* and then infected with *P. falciparum* field isolates originating from six different gametocyte carriers (designated patient, p#). **(A)** Two batches of *An. coluzzii* females were infected with parasites from p1, p2, and p3. **(B)** Three batches of *An. gambiae* females were infected with parasites from p1, p3, p4, p5, and p6. **(C)** In *An. coluzzii*, ds*EcR*-injections did not reduce cumulative egg development to the level of statistical significance (Mann-Whitney). **(D)** In *An. gambiae*, *EcR*-silencing did result in a significant reduction in cumulative egg numbers compared to controls (unpaired t-test). **(E-F)** In both species, **(E)** *An. coluzzii* and **(F)** *An. gambiae*, ds*EcR*-injections had no effect on cumulative oocyst prevalence (Fisher's Exact) or intensity (unpaired t-test and Mann-Whitney). P next to pie charts = prevalence. N = sample size. p# = parasite isolate.

Fig 2: Oocyst growth is accelerated after ds*EcR* treatment in both *An. coluzzii* and *An. gambiae*. (A) In *An. coluzzii* and **(B)** *An. gambiae*, *EcR*-depleted females had significantly larger oocysts at 8 days pIBM compared to ds*GFP* controls (Cntrl) (GLMM, LRT). Average oocyst size per midgut is shown for simplicity – analyses are based on nested data incorporating all oocyst measurements. **(C)** Representative images of oocysts found in the midgut of *An. gambiae* control and ds*EcR* females. Black dotted circles show oocyst perimeters. Scale bar = 100 µm. **(D)** At 12 days pIBM, *EcR*-silenced *An. gambiae* females had a greater number of sporozoites in their salivary glands than

controls (Mann-Whitney). Sporozoite prevalence (P) at this time point was not different (Fisher's Exact). **(E)** Expression of *Lipophorin* in ds*EcR* females was elevated relative to ds*GFP* controls (unpaired t-test). Gene expression was assessed for each batch of mosquitoes used in infections (whole body mosquito sample without head, prior to blood feeding). For applicable panels, N = sample size, or number of mosquitoes. $N_{oocysts}$ = number of individual oocyst measurements. p# = parasite isolate.

Fig 3: *P. falciparum* oocyst growth is negatively linked to egg development. (A) In *An. coluzzii* controls (ds*GFP*-injected), egg numbers are negatively associated with oocyst size, but this association is lost following ds*EcR* treatment (GLMM, LRT). **(B)** In *An. gambiae*, egg numbers are negatively associated with oocyst size in both control and ds*EcR* conditions (GLMM, LRT), but **(C)** this association differentially varies across oocyst density in control and ds*EcR* females (3-way interaction, treatment*egg#*oocyst#, GLMM, LRT, $X^2_1 = 8.57$, p = 0.003). Lines across egg numbers and oocyst size graphically represent the model-based analysis that was performed, which used nested individual oocyst measurements. Shading shows 95% confidence interval. N = sample size, or number of mosquitoes. Number of individual oocyst measurements including in analysis were: *An. coluzzii* controls = 926, *An. coluzzii* ds*EcR* = 1003, *An. gambiae* controls = 669, *An. gambiae* ds*EcR* = 760.

Fig 4: Individual *P. falciparum* isolates vary in their growth response to *EcR*-silencing. (A) For *An. coluzzii* females, both p1 and p2 parasites produced larger oocysts in *EcR*-silenced females, but p3 parasites failed to respond to ds*EcR* conditions and did

not grow larger than controls (GLMM, LRT). **(B)** For *An. gambiae*, all *P. falciparum* isolates responded to *EcR*-silencing by growing larger except for p6 parasites, which remained the same size as controls (Cntrl), even though they were fed to the same batch of mosquitoes as p5 parasites (GLMM, LRT). **(C)** Oocyst size varies significantly between *An. coluzzii* and *An. gambiae* control females infected with the same parasite isolates (p1 and p3) (GLMM, LRT), whereby oocysts are larger in *An. coluzzii* than in *An. gambiae*. **(D)** *An. coluzzii* were collected as larvae from VK5, Burkina Faso and infected with *P. falciparum* isolates p1-p6. Pre-gravid females that failed to develop eggs were less likely to become infected (pie charts, P = oocyst prevalence, Fisher's Exact), although among infected individuals, there was no difference in oocyst intensity (Mann-Whitney). **(E)** Pre-gravid females had significantly larger oocysts at 7 days pIBM compared to gravid females (GLMM, LRT). For all applicable panels, average oocyst size per midgut is shown for simplicity – analyses are based on nested data incorporating all oocyst measurements. N = sample size, or number of mosquitoes. $N_{oocysts}$ = number of individual oocyst measurements. p# = parasite isolate.

S1 Fig: Egg development for individual infections. (A-B) The effects of *EcR*-silencing on egg development across individual infections for **(A)** *An. coluzzii* (unpaired t-test and Mann-Whitney) and **(B)** *An. gambiae* (unpaired t-test and Mann-Whitney) compared to controls (Cntrl). N = sample size. p# = parasite isolate.

S2 Fig: Oocyst prevalence and intensity for individual infections. (A-B) The effects of *EcR*-silencing on the prevalence (Fisher's Exact) and intensity (unpaired t-test and

Mann-Whitney) of oocysts across individual infections for **(A)** *An. coluzzii* and **(B)** *An. gambiae,* compared to ds*GFP*-injected (Cntrl) females. P = oocyst prevalence. N = sample size. p# = parasite isolate.

S3 Fig: Sporozoite prevalence and intensity for individual infections. (A) The salivary glands of ds*EcR* and ds*GFP* (Cntrl) *An. gambiae* females were assessed at 12 days post-infected blood meal from infections with parasite isolates p1, p3, p5, and p6. The sporozoite prevalence (Fisher's Exact) and intensity (Mann-Whitney) for each infection are shown. P = sporozoite prevalence. N = sample size. p# = parasite isolate.

S4 Fig: Additional infection data for colony mosquitoes and VK5-*An. coluzzii*. (A) The majority of VK5-*An. coluzzii* females that were provided a parasite-infected (p1-p6) blood meal, failed to develop any eggs. **(B)** Oocyst prevalence (P) and intensity for individual infections with VK5-*An. coluzzii*. **(C)** Oocyst size tended to be larger in the few colony mosquitoes (ds*GFP* females from both *An. coluzzii* and *An. gambiae*) that failed to develop eggs after blood feeding (pre-gravid) when compared to their gravid counterparts (GLMM, LRT). **(D)** Mean oocyst size per VK5-*An. coluzzii* female for each infection with a different parasite isolate (P#). Mean oocyst sizes are shown for simplicity, but all analyses were done with all individual oocyst measurements nested by mosquito. N = sample size. P# = parasite isolate.

References

1. Marinotti O, Nguyen QK, Calvo E, James AA, Ribeiro JM. Microarray analysis of genes showing variable expression following a blood meal in Anopheles gambiae. Insect Mol Biol. 2005;14(4):365-73.

2. Marinotti O, Calvo E, Nguyen QK, Dissanayake S, Ribeiro JM, James AA. Genome-wide analysis of gene expression in adult Anopheles gambiae. Insect Mol Biol. 2006;15(1):1-12.

3. Roy S, Saha TT, Zou Z, Raikhel AS. Regulatory Pathways Controlling Female Insect Reproduction. Annu Rev Entomol. 2018;63:489-511.

4. Macdonald G. Epidemiological basis of malaria control. Bull World Health Organ. 1956;15(3-5):613-26.

5. WHO. Manual on Practical Entomology in Malaria. World Health Organization; 1975.

6. Vaughan JA, Noden BH, Beier JC. Population dynamics of Plasmodium falciparum sporogony in laboratory-infected Anopheles gambiae. J Parasitol. 1992;78(4):716-24.

7. Shaw WR, Holmdahl IE, Itoe MA, Werling K, Marquette M, Paton DG, et al. Multiple blood feeding in mosquitoes shortens the Plasmodium falciparum incubation

period and increases malaria transmission potential. PLoS Pathog. 2020;16(12):e1009131.

8. Guissou E, Waite JL, Jones M, Bell AS, Suh E, Yameogo KB, et al. A non-destructive sugar-feeding assay for parasite detection and estimating the extrinsic incubation period of Plasmodium falciparum in individual mosquito vectors. Sci Rep. 2021;11(1):9344.

9. Matthews J, Bethel A, Osei G. An overview of malarial Anopheles mosquito survival estimates in relation to methodology. Parasit Vectors. 2020;13(1):233.

10. Clements AN, Paterson GD. The Analysis of Mortality and Survival Rates in Wild Populations of Mosquitoes. Journal of Applied Ecology. 1981;18(2):26.

11. Costantini C, Li SG, Della Torre A, Sagnon N, Coluzzi M, Taylor CE. Density, survival and dispersal of Anopheles gambiae complex mosquitoes in a west African Sudan savanna village. Med Vet Entomol. 1996;10(3):203-19.

12. Gillies MT, Wilkes TJ. A study of the age-composition of populations of Anopheles gambiae Giles and A. funestus Giles in North-Eastern Tanzania. Bull Entomol Res. 1965;56(2):237-62.

13. Paaijmans KP, Blanford S, Bell AS, Blanford JI, Read AF, Thomas MB. Influence of climate on malaria transmission depends on daily temperature variation. Proc Natl Acad Sci U S A. 2010;107(34):15135-9.

14. Beck-Johnson LM, Nelson WA, Paaijmans KP, Read AF, Thomas MB, Bjørnstad ON. The effect of temperature on Anopheles mosquito population dynamics and the potential for malaria transmission. PLoS One. 2013;8(11):e79276.

15. Shapiro LLM, Whitehead SA, Thomas MB. Quantifying the effects of temperature on mosquito and parasite traits that determine the transmission potential of human malaria. PLoS Biol. 2017;15(10):e2003489.

16. Blanford JI, Blanford S, Crane RG, Mann ME, Paaijmans KP, Schreiber KV, et al. Implications of temperature variation for malaria parasite development across Africa. Sci Rep. 2013;3:1300.

17. Hien DF, Dabiré KR, Roche B, Diabaté A, Yerbanga RS, Cohuet A, et al. Plant-Mediated Effects on Mosquito Capacity to Transmit Human Malaria. PLoS Pathog. 2016;12(8):e1005773.

18. Shapiro LL, Murdock CC, Jacobs GR, Thomas RJ, Thomas MB. Larval food quantity affects the capacity of adult mosquitoes to transmit human malaria. Proc Biol Sci. 2016;283(1834).

19. Habtewold T, Sharma AA, Wyer CAS, Masters EKG, Windbichler N, Christophides GK. Plasmodium oocysts respond with dormancy to crowding and nutritional stress. Sci Rep. 2021;11(1):3090.

20. Kwon H, Simões ML, Reynolds RA, Dimopoulos G, Smith RC. Additional Feeding Reveals Differences in Immune Recognition and Growth of Plasmodium Parasites in the Mosquito Host. mSphere. 2021;6(2).

21. Ohm JR, Baldini F, Barreaux P, Lefevre T, Lynch PA, Suh E, et al. Rethinking the extrinsic incubation period of malaria parasites. Parasit Vectors. 2018;11(1):178.

22. Lefevre T, Ohm J, Dabiré KR, Cohuet A, Choisy M, Thomas MB, et al. Transmission traits of malaria parasites within the mosquito: Genetic variation, phenotypic plasticity, and consequences for control. Evol Appl. 2018;11(4):456-69.

23. Werling K, Shaw WR, Itoe MA, Westervelt KA, Marcenac P, Paton DG, et al. Steroid Hormone Function Controls Non-competitive Plasmodium Development in Anopheles. Cell. 2019;177(2):315-25.e14.

24. Gillies MT. The recognition of age-groups within populations of Anopheles gambiae by the pre-gravid rate and the sporozoite rate. Ann Trop Med Parasitol. 1954;48(1):58-74.

25. GILLIES MT. The pre-gravid phase of ovarian development in anopheles funestus. Ann Trop Med Parasitol. 1955;49(3):320-5.

26. Paton DG, Probst AS, Ma E, Adams KL, Shaw WR, Singh N, et al. Using an antimalarial in mosquitoes overcomes Anopheles and Plasmodium resistance to malaria control strategies. PLoS Pathog. 2022;18(6):e1010609.

27. Bolker BM, Brooks ME, Clark CJ, Geange SW, Poulsen JR, Stevens MH, et al. Generalized linear mixed models: a practical guide for ecology and evolution. Trends Ecol Evol. 2009;24(3):127-35.

28. Harrison XA, Donaldson L, Correa-Cano ME, Evans J, Fisher DN, Goodwin CED, et al. A brief introduction to mixed effects modelling and multi-model inference in ecology. PeerJ. 2018;6:e4794.

29. Hien AS, Sangaré I, Coulibaly S, Namountougou M, Paré-Toé L, Ouédraogo AG, et al. Parasitological Indices of Malaria Transmission in Children under Fifteen Years in Two Ecoepidemiological Zones in Southwestern Burkina Faso. J Trop Med. 2017;2017:1507829.

30. Epopa PS, Collins CM, North A, Millogo AA, Benedict MQ, Tripet F, et al. Seasonal malaria vector and transmission dynamics in western Burkina Faso. Malar J. 2019;18(1):113.

31. Kaneko O, Kimura M, Kawamoto F, Ferreira MU, Tanabe K. Plasmodium falciparum: allelic variation in the merozoite surface protein 1 gene in wild isolates from southern Vietnam. Exp Parasitol. 1997;86(1):45-57.

32. Scott TW, Takken W. Feeding strategies of anthropophilic mosquitoes result in increased risk of pathogen transmission. Trends Parasitol. 2012;28(3):114-21.

33. Lyimo EO, Takken W. Effects of adult body size on fecundity and the pre-gravid rate of Anopheles gambiae females in Tanzania. Med Vet Entomol. 1993;7(4):328-32.

34. Takken W, Klowden MJ, Chambers GM. Effect of body size on host seeking and blood meal utilization in Anopheles gambiae sensu stricto (Diptera: Culicidae): the disadvantage of being small. J Med Entomol. 1998;35(5):639-45.

35. Ye YH, Chenoweth SF, Carrasco AM, Allen SL, Frentiu FD, van den Hurk AF, et al. Evolutionary potential of the extrinsic incubation period of dengue virus in Aedes aegypti. Evolution. 2016;70(11):2459-69.

36. Neafsey DE, Schaffner SF, Volkman SK, Park D, Montgomery P, Milner DA, et al. Genome-wide SNP genotyping highlights the role of natural selection in Plasmodium falciparum population divergence. Genome Biol. 2008;9(12):R171.

37. Volkman SK, Sabeti PC, DeCaprio D, Neafsey DE, Schaffner SF, Milner DA, et al. A genome-wide map of diversity in Plasmodium falciparum. Nat Genet. 2007;39(1):113-9.

38. Manske M, Miotto O, Campino S, Auburn S, Almagro-Garcia J, Maslen G, et al. Analysis of Plasmodium falciparum diversity in natural infections by deep sequencing. Nature. 2012;487(7407):375-9.

39. WHO. World Malaria Report. World Health Organization; 2019.

40. Sinka ME, Bangs MJ, Manguin S, Rubio-Palis Y, Chareonviriyaphap T, Coetzee M, et al. A global map of dominant malaria vectors. Parasit Vectors. 2012;5:69.

41. Crawford JE, Riehle MM, Markianos K, Bischoff E, Guelbeogo WM, Gneme A, et al. Evolution of GOUNDRY, a cryptic subgroup of Anopheles gambiae s.l., and its impact on susceptibility to Plasmodium infection. Mol Ecol. 2016;25(7):1494-510.

42. Wiebe A, Longbottom J, Gleave K, Shearer FM, Sinka ME, Massey NC, et al. Geographical distributions of African malaria vector sibling species and evidence for insecticide resistance. Malar J. 2017;16(1):85.

43. Nguyen PL, Vantaux A, Hien DF, Dabiré KR, Yameogo BK, Gouagna LC, et al. No evidence for manipulation of Anopheles gambiae, An. coluzzii and An. arabiensis host preference by Plasmodium falciparum. Sci Rep. 2017;7(1):9415.

44. Santolamazza F, Mancini E, Simard F, Qi Y, Tu Z, della Torre A. Insertion polymorphisms of SINE200 retrotransposons within speciation islands of Anopheles gambiae molecular forms. Malar J. 2008;7:163.

45. Baldini F, Gabrieli P, South A, Valim C, Mancini F, Catteruccia F. The interaction between a sexually transferred steroid hormone and a female protein regulates oogenesis in the malaria mosquito Anopheles gambiae. PLoS Biol. 2013;11(10):e1001695.

46. Blandin S, Moita LF, Köcher T, Wilm M, Kafatos FC, Levashina EA. Reverse genetics in the mosquito Anopheles gambiae: targeted disruption of the Defensin gene. EMBO Rep. 2002;3(9):852-6.

47. Rogers DW, Whitten MM, Thailayil J, Soichot J, Levashina EA, Catteruccia F. Molecular and cellular components of the mating machinery in Anopheles gambiae females. Proc Natl Acad Sci U S A. 2008;105(49):19390-5.

48. Harris C, Morlais I, Churcher TS, Awono-Ambene P, Gouagna LC, Dabire RK, et al. Plasmodium falciparum produce lower infection intensities in local versus foreign Anopheles gambiae populations. PLoS One. 2012;7(1):e30849.

49. Sangare I, Dabire R, Yameogo B, Da DF, Michalakis Y, Cohuet A. Stress dependent infection cost of the human malaria agent Plasmodium falciparum on its natural vector Anopheles coluzzii. Infect Genet Evol. 2014;25:57-65.

50. Bousema T, Dinglasan RR, Morlais I, Gouagna LC, van Warmerdam T, Awono-Ambene PH, et al. Mosquito feeding assays to determine the infectiousness of naturally infected Plasmodium falciparum gametocyte carriers. PLoS One. 2012;7(8):e42821.

51. Vantaux A, Ouattarra I, Lefèvre T, Dabiré KR. Effects of larvicidal and larval nutritional stresses on Anopheles gambiae development, survival and competence for Plasmodium falciparum. Parasit Vectors. 2016;9:226.

52. Schindelin J, Arganda-Carreras I, Frise E, Kaynig V, Longair M, Pietzsch T, et al. Fiji: an open-source platform for biological-image analysis. Nat Methods. 2012;9(7):676-82.

53. Snounou G, Viriyakosol S, Zhu XP, Jarra W, Pinheiro L, do Rosario VE, et al. High sensitivity of detection of human malaria parasites by the use of nested polymerase chain reaction. Mol Biochem Parasitol. 1993;61(2):315-20.

Click here to access/download;Figure;Figure 1.pdf

Figure 1

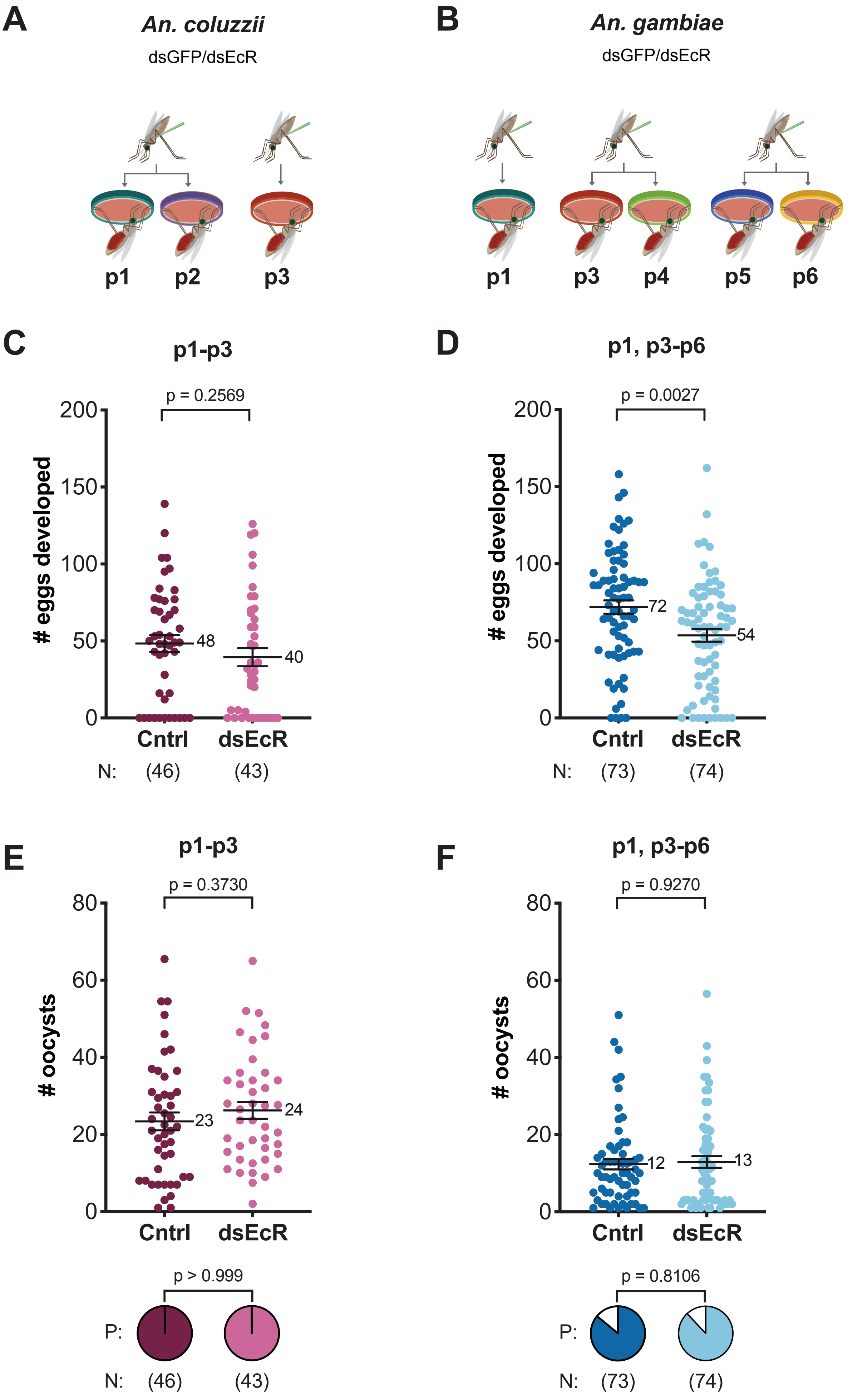

Figure 2

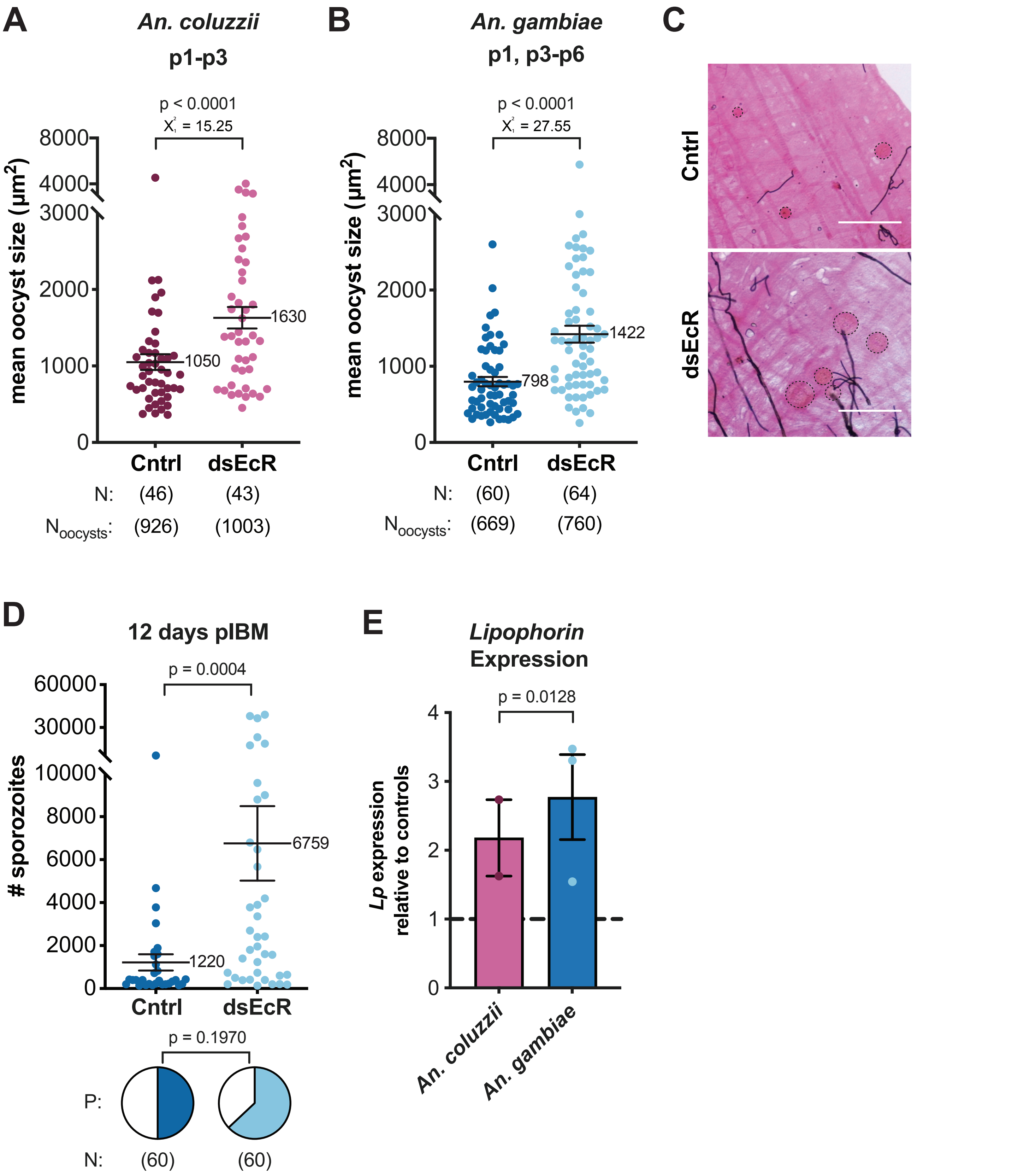
A
An. coluzzii
p1-p3
p < 0.0001
X = 15.25
mean oocyst size (µm²)
8000
4000
3000
2000
1000
0
1050
1630
Cntrl
dsEcR
N: (46) (43)
Noocysts: (926) (1003)
B
An. gambiae
p1, p3-p6
p < 0.0001
X = 27.55
798
1422
N: (60) (64)
Noocysts: (669) (760)
C
Cntrl
dsEcR
D
12 days pIBM
p = 0.0004
# sporozoites
60000
30000
10000
8000
6000
4000
2000
1220
6759
p = 0.1970
P:
N: (60) (60)
E
Lipophorin
Expression
p = 0.0128
Lp expression
relative to controls
An. coluzzii
An. gambiae

Figure 3

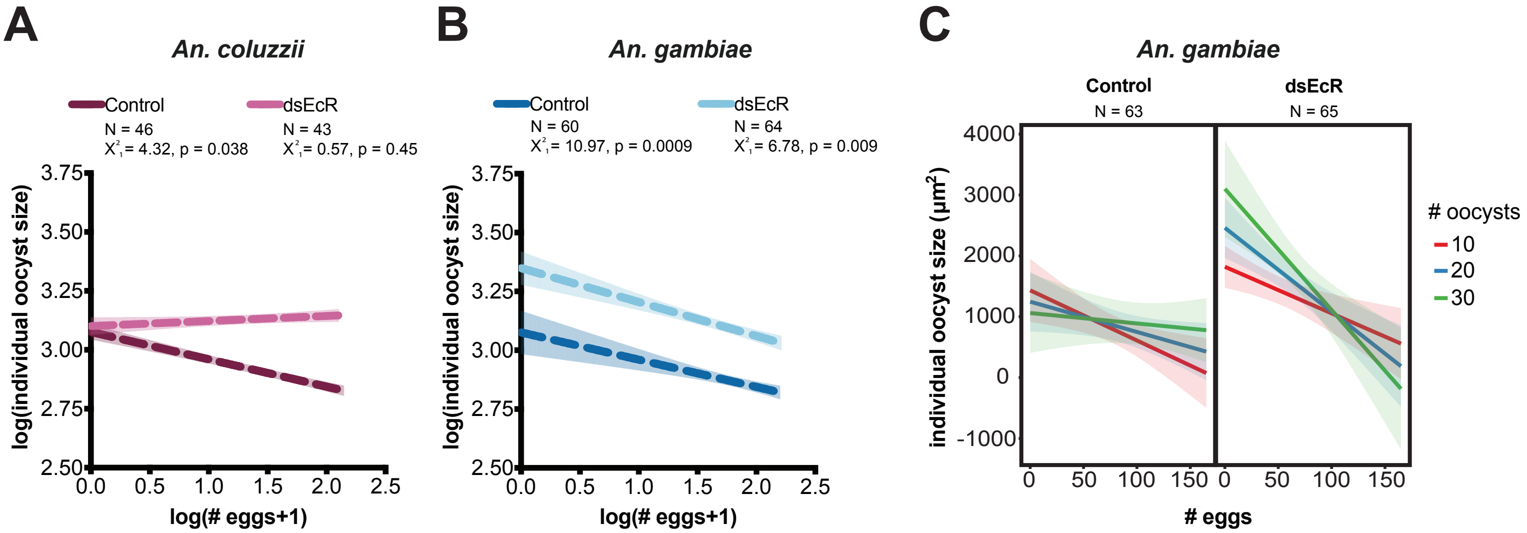
A
An. coluzzii
Control
N = 46
X^2_1 = 4.32, p = 0.038
dsEcR
N = 43
X^2_1 = 0.57, p = 0.45
log(individual oocyst size)
log(# eggs+1)
B
An. gambiae
Control
N = 60
X^2_1 = 10.97, p = 0.0009
dsEcR
N = 64
X^2_1 = 6.78, p = 0.009
log(individual oocyst size)
log(# eggs+1)
C
An. gambiae
Control
N = 63
dsEcR
N = 65
individual oocyst size (μm²)
oocysts
10
20
30
eggs

Figure 4 Click here to access/download;Figure;Figure 4.pdf

Figure 4

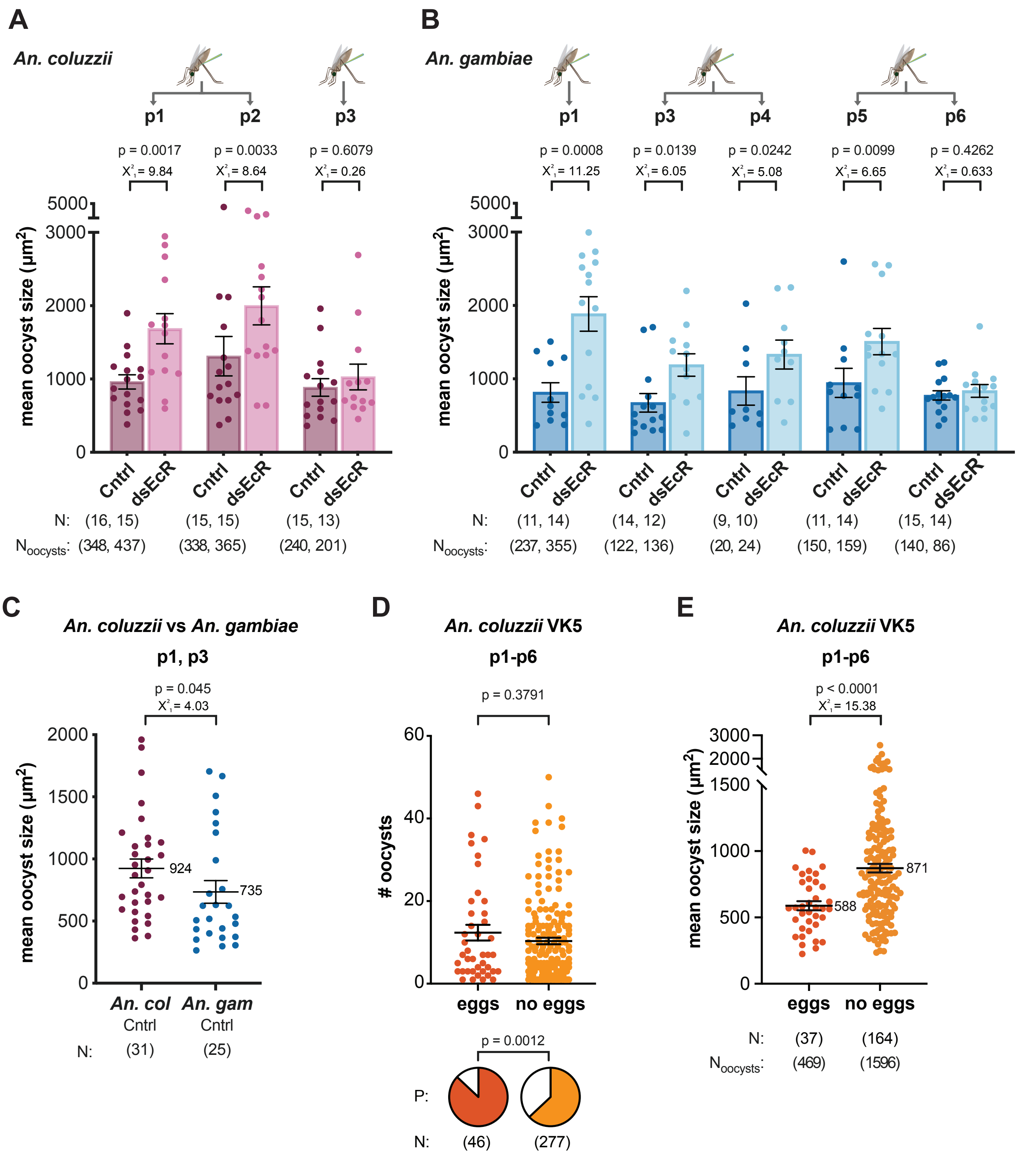

Click here to access/download
Supporting Information
Supplementary Information.pdf
