# Supplementary material for: Development of circulating isolates of Plasmodium falciparum is accelerated in Anopheles vectors with reduced reproductive output
Source: PLoS Negl Trop Dis. 2024 Jan 11;18(1):e0011890. doi: 10.1371/journal.pntd.0011890 (PMC10807765; doi:10.1371/journal.pntd.0011890)
Supplement: Attachment — Submitted filename: Letter_Response_to_Reviewers_FINAL.pdf [file pntd.0011890.s010.pdf]

Responses to Reviewer's Comments

Methods:

Reviewer #1: No concerns.

Reviewer #2: Study design is appropriated and the data supports the hypothesis presented. I don't see any statistical issues in the manuscript.
We are glad that this reviewer finds our study design and statistical interpretations appropriate and supportive of the hypotheses presented.

Reviewer #3: Objectives clearly articulated. I think in some cases the power is lacking to address some of the hypotheses but this is a restriction that comes with this type of study, rather than poor design. I have commented on the text for such occasions.
We agree with the reviewer that in some cases our datasets lack the power that we ideally would have wanted to fully address all our hypotheses, but as the reviewer also points out, this is an inherent limitation that comes with this type of field study. We hope that our hypotheses presented here can be further tested and scrutinized with additional, future field studies. In response to this reviewer's specific concerns noted in the text, we have modified the manuscript where appropriate and removed statements that didn't have enough statistical power to fully support them. We believe our modifications have made our conclusions and interpretations even more reflective of the data itself. We have listed our specific changes to the manuscript below in response to the specific concerns of the reviewer.

Results:

Reviewer #1: Generally well-presented, only minor comment regarding the figures to improve clarity.
We believe we have addressed all points raised by this reviewer in the provided commentary.

Reviewer #2: The hypothesis is well supported by the dat, the results are clear. Figures and diagrams are very well put together.
We appreciate that this reviewer found our study results to be clear and the figures well put together.

Reviewer #3: The figures are of good quality and are explanatory. The results are hard to follow but again, this is because they are complex and sometimes contradictory, depending on either genotype, batch or condition. I think a schematic figure summarising findings might be helpful.
We thank the reviewer for commenting that our figures are "of good quality" and "explanatory." Due to the complexity of the experimental set-up involving multiple different parasite isolates and mosquito batches, we had provided the schematic of the experimental set-up in Fig 1A-B. We also agree that the results of the study can be complex, and we think the changes we have made to the results, discussion, and methods sections, as suggested by the reviewers, have improved the clarity of the study's findings for the reader. Additionally, as we have assessed multiple factors across several batches/species of mosquitoes and parasites (including egg development, oocyst prevalence, oocyst number, oocyst size, and in some cases sporozoite prevalence and number), we don't consider a schematic encompassing all these variables

would be particularly helpful for the reader. The major take-away from this study – that in field settings, parasite growth is accelerated when mosquito reproduction is reduced – we believe is captured in our title and abstract.

Conclusions:

Reviewer #1: The authors tackle an important question as to how lab observations equate to more natural infection settings. The manuscript can be improved by better articulating how potential differences may result between this study and those previously published from the group.

From this study, we were excited to see that the main finding from our previous laboratory work – impaired 20E signaling and reduced reproductive investment is linked to faster parasite development – was recapitulated in our present study in a more natural infection setting. This finding demonstrates that the interaction between egg development and oocyst development uncovered in the laboratory is also present in field settings. On the other hand, we did find one key difference between the results of this study and previously published work, whereby ds*EcR* treatment reduced oocyst numbers in our previous laboratory study but not in the present study performed with field-derived mosquitoes and parasites. We had noted in the discussion that this discrepancy may be due to differences in overall oocyst load (which was higher in the lab than in the field experiments) and that further work is needed to understand how oocyst density might be a contributing factor to these interactions. Now, we have also further expanded on this aspect of the discussion by mentioning that this difference between laboratory and field findings might also be due to the genetic differences in the parasites and mosquitoes used in the previous laboratory studies compared to the present work. Understanding more precisely how genetic factors might affect both oocyst numbers and oocyst growth in interaction with mosquito reproductive processes will be interesting to further pursue in future studies.

Reviewer #2: In the present manuscript the authors investigate if that relationship between reproduction and malaria transmission is relevant to endemic regions that present different parasite genotypes and mosquito species. The work is very relevant and well put together.
The differences found between colluzzii and gambiae are very interesting and point that the role of 20E might not be only related to oogenesis in gambiae.
Very interesting the data with the pre-gravid females collected from the field. I wondered if the presumably different microbiota from the field collected and the lab colonies could play a role in the percentage of pre-gravid females. Please address this in the discussion.

The reviewer brings up a good point in that the mosquito microbiota may play a role in determining pre-gravid rates in the field (vs what we observe in the lab). We have added a comment to the discussion section - noting that in addition to teneral reserves, pre-gravid rates may also be affected by the microbiota, as this could affect nutrient processing (and therefore egg development). The link between mosquito microbiota and egg development has been demonstrated by others in past studies and these have been added as references (Coon et al, Parasit Vectors. 2016.; Gimonneau et al, Infect Genet Evol. 2014), in addition to a reference that details the microbiota of *An. gambiae* and *An. coluzzii* through different life stages (Gendrin et al, Nat Commun. 2015).

Reviewer #3: Yes i think the conclusions are supported by the data presented and the public health relevance is addressed. Limitations of the analysis and caveats on the interpretation are usually well considered, although there is the impression that the language leans to emphasise one interpretation over another when the data might be less conclusive - for example 'there is a trend' when not significant but in line with the preferred hypothesis and 'no effect' when not significant and not in line with preferred hypothesis (see also 'partially contrast').
We appreciate that the reviewer found our conclusions to be supported by the data and also noticed that we comment on the limitations and caveats of the study when appropriate. In order to address the reviewer's concern that at times our language leans to emphasize certain data interpretations over others, we have adjusted the manuscript in accordance with their in-text comments/suggestions and appreciate their taking the time to highlight these specific instances. The specific point-by-point responses are below, but for example, we have decided to remove such instances where we refer to "trends" or "marginal effects" in the data, as were found in the results section lines 122, lines 140-144, and lines 247-248 in the original submission. We have also removed words like "slightly" (line 241) and added clarification to sections noting "partial" differences between lab and field studies. We hope that removing and modifying these such words/comments will allow for a more fair and unbiased interpretation of the data by a reader.

Editorial and Data Presentation Modifications:
Reviewer #1: (No Response)

Reviewer #2: Line 41 – "when we disrupt signally" - replace by signaling.
Line 62 – phrase reads strange to me – replace by "female feeds on blood again".
Both of these edits have been made in the revised manuscript.

Reviewer #3: I find the short title provided by the authors "Plasmodium growth is linked to mosquito oogenesis in field settings" to be a fairer, and more accurate reflection of the experimental findings presented in this paper compared to the current working title. I would ask the authors to consider using the shorter form, or a modified version thereof.
It is not clear to us which specific aspect of the current title the reviewer took issue with, but in response to their comment we have modified the working title, aiming to capture more of the data presented in the manuscript. The new title we propose is: "Development of circulating isolates of *Plasmodium falciparum* is accelerated in *Anopheles* vectors with reduced reproductive output." We feel that this title is reflective of the data shared for ds*EcR* treatments in *An. gambiae* and *An. coluzzii* as well as the pre-gravid VK5 individuals. We hope the reviewer also finds this title a more accurate reflection of the findings.

Summary and General Comments:
Reviewer #1: (No Response)

Reviewer #2: Previously this group had reported that mosquito reproduction is negatively correlated to P.falciparum oocyst growth rates. That implies that mosquitoes that do not develop eggs can transmit parasites sooner, representing an advantage to the parasite considering the

lifespan of the mosquito. I have some small comments and I believe minor editing should be done before publication, but the manuscript is well written, the hypothesis is supported by the data and the work represents an important advancement in our understanding of the trade of between reproduction and parasite growth in the field.
We thank the reviewer for commenting on the importance of our study and findings. We believe we have addressed this reviewer's comments and suggested edits in the revised manuscript and thank them for taking the time to review our study.

Reviewer #3: This is an important piece of work, looking at the interaction between oogenesis and parasite development. While it has long been known that there are trade offs in investment in immunity, which can affect parasite development and numbers, and reproduction, these authors previously showed in lab mosquitoes that the correlation was not necessarily as expected; they interfered with oogenesis by disrupting ecdysone signalling and this resulted in faster oocyst development.
In this study they expand this study to field caught (or progeny of field caught mosquitoes) to see how true this holds in nature. Not all the previous findings can be recapitulated, which is fine and some what to be expected - if things always held true there would be no point testing in the field first. These findings will be of interest to a wide range of researchers in the field of vector control and insect physiology. As mentioned above, I do think there are some sections where findings are a little overinterpreted and the language could be modified. Some of the generality of the findings is limited in power by the availability of all parasite and vector genotype combinations but this is still a significant amount of work and commendable.
Rather than list all my points here I have included an annotated copy of the manuscript and below i list just a few of the more significant technical comments/queries:

There is a general incongruity in the level of statistical description and/or language that is used in the different sections
We agree with the reviewer that there was incongruity in how statistics were reported in the different sections of the results. In only one section (titled, "Parasite isolates vary in their growth response to impaired 20E signaling"), we had statistical results reported in the main text, while in all other sections statistics were reported in the figures and figure legends. In order to be consistent in style throughout, we have now moved all descriptions of statistical analyses into the figures/figure legends. Furthermore, we have also removed comments about the data that are not fully backed by statistical findings, including comments on "trends, or marginal effects" to be fairer and more unbiased in the language we use to describe the data throughout the manuscript.

Line 199 you still cannot exclude batch effects (on the parasite side) here though, can you? presumably you don't have separate batches of the parasite? And p6 was not compared across mosquito batches, or across mosquito species, was it?
We realize that this part was confusing for the reviewers, and we have therefore provided a more detailed explanation of the experimental set up in the initial section as well as here. Briefly, p5 and p6 are two different parasite isolates collected on the same day but from two different individuals. These parasites were provided to the same batch of *An. gambiae* mosquitoes

(same generation, injected with dsRNA on the same day) via membrane-feeding assay. From MSP1-typing, we later found that these parasite isolates are genetically different from one another because they differ in the number and combination of genotypes they contain. Because these parasite isolates responded differently to EcR-silencing in the same batch of mosquitoes, this leads to our hypothesis that different parasite genotypes may interact differentially with the processes that lead to mosquito egg development.

P5 and P6 were not compared across mosquito batches or species, because we only had *An. gambiae* available for this infection date, and critically, the parasites cannot be saved and used for mosquito infections on later days. It is extremely challenging (and takes a long time) to culture-adapt field collected parasites, and parasite-infected blood will not remain infectious over time. Given these factors, we were limited in the number of comparisons were could feasibly make in this study alone. We have now specified that parasites were used in infection without culture adaptation and have added some additional information in the materials and methods sections.

Line 247 re: Fig4E oocyst sizes in pre-gravid versus gravid: this seems confounded by the fact that An col and An gamb are mixed here. An gam apparently had zero 'pre-gravid' females, so there can be no signal here. If you remove An gambiae and look only at An coluzzi, is there a significant difference?
There is only a single *An. gambiae* female who failed to develop eggs. Following this reviewer's questions, we removed that single *An. gambiae* from the data set, and we do not detect a significant difference in oocyst size based on egg prevalence in *An. coluzzii* alone. We realized that these data were very uneven in terms of number of females who failed to develop eggs, which was one reason we sought to address the question of pre gravid status in field caught mosquitoes as opposed to the colony females. For these reasons, we have decided to remove figure panel S4C from the original submission.

Line 274 re: interactions at low density vs high density: this seems circular to me. Accepting, for a minute, the hypothesis of limiting available resources - the relative decrease per oocyst is the same in both condition. In those where there is higher density of oocysts and therefore even less available resources to start with, wouldn't one expect the effect to be further exacerbated at this density. The point made here seems stretched and thin.
In control *An. gambiae* (left panel in Figure 3C), our data show that the negative effect of egg number on oocyst size is stronger at low oocyst densities than at high densities. Since we did not directly quantify the level of available resources in these mosquitoes, the reviewer is correct to highlight that the connection established here between the pattern observed in this figure and possible availability of resources is thin. Accordingly, we have rephrased as follows: "In the case of control *An. gambiae*, the impact of oocyst density on this association suggests that at low-density infections, any alteration in the pathways guiding egg development has a more pronounced effect on oocyst growth compared to high-density infections."

Line 278 'perturbs the effect of parasite density on egg-oocyst interactions' there is a lot in this statement - at first reading one could take this to mean there is no longer an effect but in fact,

the effect of density is still there, but the order of strength of correlation at the different densities is now reversed, is it not? i.e. in the control condition the negative correlation is highest (slope is steepest) at low densities, whereas in the dsEcR condition the negative correlation is highest at high densities.
The reviewer is correct – this statement was too vague and could lead to misinterpretation by the reader. As a result we have expanded the discussion of these data to the following: "Disruption of the 20E signaling pathway perturbs the effect of parasite density on these egg by oocyst size interactions. This time, the most pronounced negative influence of egg number on oocyst growth is observed at high oocyst densities. Proximal mechanisms explaining these results are currently unknown. The relationships among infection, reproduction, nutrient intake, and the overall condition of the host are complex and future studies would be required to quantify, for example, the resource levels in control and ds*EcR* mosquitoes with high vs low oocyst densities."

Line 324 (and elsewhere) Re: lack of ability to validate EcR knockdown after dsEcR injection. It would would be nice to get more clarity on this (here or in Methods). Could you not look at EcR transcript levels after bloodfeeding? Did you look at EcR transcript levels and were not able to detect transcripts? Or able to detect transcripts but no knockdown?
The expression levels of *EcR* in mosquito midguts are low and variable, making it difficult to reliably establish occurrence of knock-down of this gene. Therefore, in our hands a better measure of knock-down efficacy is to look at genes controlled by this nuclear receptor. Given that we knew *Lp* is upregulated when *EcR* is silenced, we used upregulation of this gene as a reliable marker for knock-down. We have now rephrased the sentence in the manuscript clarifying this point.

Line 344 re: need to adapt to sympatric species. This is a lot of speculation and, interesting as it is, has little data to support. You might at least suggest for the reader the next steps to prove it
We agree with the reviewer that this hypothesis is highly speculative and following the reviewer's suggestion we have added a sentence to suggest what is needed to support it.

Line 351 this is a great point, but doesn't it argue against the hypothesis just made (344-347)?
The reviewer raises an important point and one that we abundantly debated when we first got these data and during the preparation of the original manuscript. We believe that the reasons why we didn't observe any isolate-driven effects on oocyst size in ds*GFP* or VK5 mosquitoes, while we did in ds*EcR* females, is that when we disrupt 20E signaling we are pushing mosquito physiology outside its natural range, such that we can uncover responses/interactions that might not be detectable in unmanipulated mosquitoes. This effect may be magnified by the clonality of infection, as polyclonal infections (as present in this study) may mask differences in parasite growth in normal conditions but not when growth is favored and some genotypes respond differentially to the stimuli. We have now added a sentence in the text to clarify this point.

Line 459 out of interest, what was the justification for only taking children?
There are two main reasons for recruiting volunteers between the ages of 5-13 in this study. Firstly, from a biological standpoint, it is generally observed that children of this age range

exhibit significantly higher gametocyte prevalence and density compared to individuals older than 15 years. Several references support this observation (e.g. Churcher et al. 2013 Elife DOI: 10.7554/eLife.00626; Goncalves et al. 2017 Nat Com DOI:10.1038/s41467-017-01270-4). Collecting blood samples from individuals below the age of 5 poses ethical and technical challenges. Secondly, for logistical reasons related to parasite screening, it's efficient to collaborate with teachers and nurses in elementary schools. This approach allows us to work with children who are already grouped together, streamlining the screening process. Plus, activities to raise awareness about malaria and its control measures are being conducted in collaboration with the teaching teams on these occasions. This method proves to be more practical than navigating from house to house in the villages and asking for a meeting in the village square. Furthermore, as noted in the methods, any person who is found to be positive for malaria is offered treatment, regardless of their participation in the study, ensuring that studies designed in this way can have a positive impact on the local community. Clarification regarding the use of children in the study was added to the methods section.

Other suggested edits from Reviewer 3 provided directly in manuscript text:

Line 57: “helpful to define 'rapidly' - it takes several hours and this would be slow for many - it's certainly much slower compared to gametogenesis”
We removed the word “rapidly” and modified the sentence to be more specific and also added a relevant reference on the timing of ookinete formation.

Line 60: “through asexual replication (sporogony)”
Removed “(sporogony)” from previous sentence and incorporated reviewer’s above suggestion into next sentence, now reading, “Oocysts subsequently grow over several days and through asexual replication (sporogony) produce thousands of sporozoites…”

Line 70: corresponds to – “constitutes a significant fraction of”
This sentence has been modified according to the reviewer’s suggestion and now reads, “This time constitutes a significant fraction of the rather limited lifespan…”

Line 119: “what is the justification for splitting the parasite isolates this way?”
We do not have a scientific reason for splitting the parasite isolates in this way. We would have liked to test all mosquitoes with all parasite isolates, but given the challenges of working with non-lab adapted organisms (field-derived mosquitoes and human collected parasite isolates), we had to work flexibly with our experiments and performed all the combinations we could with the material that was available at the time of experimentation.

From the parasite side, these are not cultured parasites, so infection experiments can only take place on specific days when study participants travel on-site to donate blood. Furthermore, ~500uL of gametocytic blood must be introduced into each membrane feeder used to blood feed mosquitoes, so in order to minimize the volume of blood collected from the young study participants, we must make some limitations on our experimental set-up and often cannot perform all comparisons of interest simultaneously. Additionally, parasites cannot be saved for mosquito

infections on future days. These challenges needed to be further coordinated with the mosquito side as well, and these field-derived mosquitoes can vary in number of progeny produced each generation, limiting what might be available for experimental use at a given time.

To highlight the reasoning behind our experimental design and splitting the parasite isolates this way, we have added clarification to the beginning of the results section.

Line 122: only marginally affected – "we saw no significant effect of dsEcR treatment"
We have removed "only marginally" and replaced with "eggs were not significantly affected"

Line 125: "why 'partially' ?"
We indicated "partially" because both here and in past laboratory experiments, we observed that ds*EcR* treatment does not impact the prevalence of infection. This finding is consistent in both settings. On the other hand, we previously saw that ds*EcR* treatment reduced oocysts numbers in the laboratory, but saw no effect here. The "partially" is to indicate that the prevalence data was consistent but intensity data was not. We have added in clarification into the text to highlight this distinction.

Line 140: "....and would explain why we observe this phenomenon in the absence of any difference in oocyst numbers between conditions"
We have incorporated the reviewer's suggested text as, "so it is likely that this increase in sporozoites reflects faster parasite development also considering the absence of any difference in oocyst numbers between conditions."

Line 141: this is a bit weak. Can you not do ANOVA or some other more appropriate stats test?
Following the reviewer's suggestion, we performed an ANOVA on these data and the test result was significant ($p = 0.0131$, in line with the data and analysis shown in Fig 2D), but when we went to the next step and performed the pairwise tests with multiple test corrections, none of the individual comparisons were significant (Dunn's multiple comparison tests, $p > 0.05$). With so few n's per condition, there is simply not enough statistical power when the data is broken down by infection/gam carrier. As a result, we have decided to remove this comment regarding trends across the individual infections from the manuscript text.

Line 142: sporozoite prevalence – "i would argue this is even weaker! and not statistically significant under any test applied"
In response to the reviewer's comment on the interpretation of the sporozoite prevalence data, we have decided to remove the sentence of concern from the manuscript.

Line 148-151: "did you look at other genes?"
We did not evaluate transcript levels of other metabolic or immune-related genes. Our previous study using laboratory mosquitoes indicated that lipids and Lp specifically were key factors driving accelerated oocyst growth in mosquitoes with disrupted 20E signaling. Therefore, for this study, we wanted to focus on Lp expression to see if it was consistent with our observations in the lab. It is expected that other genes are also involved in the phenotypes that we observed,

but given the key role that Lp has previously been shown to play in oocyst development by ourselves and others, this was a key gene we wanted to assess in these mosquitoes, especially as we didn't have an abundance of material for testing large panels of other genes.

Line 160: suggesting – "indicating"
We have made this suggested change to the manuscript – changing "suggesting" to "indicating" in the location cited by the reviewer.

Line 189-190: "this level of statistical description is in stark contrast to the earlier section where it was "but there's a trend" (and no statistical descriptors)"
This comment has now been addressed above. Briefly, we have moved all statistical descriptors to the figures/figure legends to be consistent throughout the results section. We have also removed comments on the data that reflect only "trends" or "marginal effects" to be faired in our presentation of findings.

Line 205-208: "the caveat is welcome, but as you say it is necessitated by not having many parasite cultures tested across both species. So all the more reason for stating earlier on why this was so"
We have now addressed this point above.

Table 1 – "formatting issue"
Formatting issue arose during conversion from .doc to .pdf, we will ensure this issue does not persist.

Line 232: "non-mated?"
All females in these experiments were non-mated (virgin), because we wanted to be able to control for mating status and not introduce that as another variable which can affect mosquito-parasite interactions. Additionally, we needed the mosquitoes to retain their eggs so we could perform our egg vs oocyst analysis for each individual female. If the females were mated, they would oviposit their eggs around the cage (on sugar pad, humidity cup), and we would not be able to link egg numbers to oocyst numbers in each individual. Putting females into individual oviposition cups is risky/challenging when working with parasite-infected mosquitoes and also increases the risk of mosquitoes dying/drowning. We have added this clarification into the materials and methods section, indicating that all females in this study were intentionally kept virgin.

Line 241: "slightly" is highlighted
We have removed the word "slightly" from this sentence, so as not to skew the reader's interpretation of the results by potentially minimizing this effect.

Line 246: "could you split this up, by parasite genotype, as you did in panels 4A and 4B? At least in a supp figure? Oh wait, you did do this in FigS4D"
We believe the reviewer's initial question is addressed by Figure S4D, now S4C.

Line 312: “partly” is highlighted

To clarify this sentence and remove the word “partly,” the sentence in question has been changed to read, “oocyst intensity was not affected by *EcR* silencing in either *An. coluzzii* or *An. gambiae* females (**Fig 1E-F**). This is in contrast to our previous study where we found a reduction in parasite numbers during the ookinete-oocyst transition”

Line 452: for the cost of including a small table, and the benefits in potential replication of findings, it is definitely worth including a small table of primers in the Supp Info

We agree and have now added the qPCR primer sequences for Lp and Rpl19 into the corresponding methods section. We have also added the sequences of the dsRNA constructs used for both EcR silencing and GFP control treatments into the methods section as well. Primer sequences used for other analyses in the study (eg MSP1-typing) were already present in the methods section.

Additional, recent references were added to the introduction/discussion where appropriate. They are:

- Guissou E, Da DF, Hien DFS, Yameogo KB, Yerbanga SR, Ouédraogo GA, et al. Intervention reducing malaria parasite load in vector mosquitoes: No impact on Plasmodium falciparum extrinsic incubation period and the survival of Anopheles gambiae. PLoS Pathog. 2023;19(5):e1011084.
- Lambert B, North A, Godfray H.C.J. A Meta-analysis of Longevity Estimates of Mosquito Vectors of Disease. bioRxiv. 2022.
